# Growth Media Conditions Influence the Secretion Route and Release Levels of Engineered Extracellular Vesicles

**DOI:** 10.1002/adhm.202101658

**Published:** 2021-11-21

**Authors:** Jeremy P. Bost, Osama Saher, Daniel Hagey, Doste R. Mamand, Xiuming Liang, Wenyi Zheng, Giulia Corso, Oskar Gustafsson, André Görgens, CI Edvard Smith, Rula Zain, Samir El Andaloussi, Dhanu Gupta

**Affiliations:** ^1^ Department of Laboratory Medicine Karolinska Institutet Huddinge 14152 Sweden; ^2^ Department of Pharmaceutics and Industrial Pharmacy Faculty of Pharmacy Cairo University Cairo 11562 Egypt; ^3^ Department of Biology Faculty of Science Cihan University‐Erbil Arbil 5XC8+WV Iraq; ^4^ Centre for Rare Diseases Department of Clinical Genetics Karolinska University Hospital Stockholm SE‐171 76 Sweden

**Keywords:** ectosomes, EV production, exocytosis, exosomes, extracellular vesicles

## Abstract

Extracellular vesicles (EVs) are nanosized cell‐derived vesicles produced by all cells, which provide a route of intercellular communication by transmitting biological cargo. While EVs offer promise as therapeutic agents, the molecular mechanisms of EV biogenesis are not yet fully elucidated, in part due to the concurrence of numerous interwoven pathways which give rise to heterogenous EV populations in vitro. The equilibrium between the EV‐producing pathways is heavily influenced by factors in the extracellular environment, in such a way that can be taken advantage of to boost production of engineered EVs. In this study, a quantifiable EV‐engineering approach is used to investigate how different cell media conditions alter EV production. The presence of serum, exogenous EVs, and other signaling factors in cell media alters EV production at the physical, molecular, and transcriptional levels. Further, it is demonstrated that the ceramide‐dependent EV biogenesis route is the major pathway to production of engineered EVs during optimized EV‐production. These findings suggest a novel understanding to the mechanisms underlying EV production in cell culture which can be applied to develop advanced EV production methods.

## Graphical abstract

Components of the cellular environment determine the quantity produced and the biogenesis of engineered extracellular vesicles (EVs). Cell media that contain serum alter EV production on several levels, including the biomolecular and transcriptional levels. Using serum‐free Opti‐MEM as an EV‐production media increases the quantity of engineered EVs produced in a ceramide‐dependent manner.

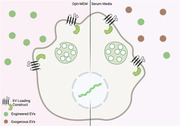

## 1 Introduction

Extracellular vesicles (EVs) are naturally occurring, nanosized cell‐derived vesicles found in all bodily fluids^[^ ^1^, ^2^, ^3^ ^]^ and are capable of providing a route of intercellular communication by transmitting biological signals between cells in vitro and in vivo.^[^ ^4^ ^]^ EV‐mediated communication is important to homeostasis maintenance, but it can also pathologically promote the intercellular spread of disease.^[^ ^5^, ^6^ ^]^ Additionally, EVs isolated from pathological states contain diagnostic information and biomarkers. Focus is growing in the area of designing and using EVs to suppress diseases such as cancer.^[^ ^7^, ^8^ ^]^ The potential advantages of EV therapeutics include their endogenous ability to target specific tissues and the low levels of toxicity and immunogenicity they exhibit.^[^ ^9^, ^10^ ^]^

Functional EVs can be divided into exosomes or ectosomes based on their biogenesis pathways.^[^ ^11^ ^]^ The two subgroups have become the focus of research groups aiming to exploit the endogenous capabilities of EVs to transfer cargo between cells. Ectosomes are formed via outward budding of the plasma membrane and may be larger in diameter than exosomes. Exosomes are generally 40–200 nm in diameter and originate from the endocytic pathway in a process which involves the endosomal membrane budding inward to form several intra‐luminal vesicles (ILVs) within a single endosome. This endosome then becomes classified as a multivesicular body (MVB) and is transported toward the cell periphery to fuse back to the plasma membrane to exocytose the ILVs, which then become exosomes in the extracellular environment.^[^ ^12^ ^]^

Both ectosomes and exosomes have been shown to carry a variety of cargo including nucleic acids, lipids, and proteins.^[^ ^13^, ^14^ ^]^ Protein cargo of EVs may be present in the lumen or they may be membrane‐associated. Membrane‐associated proteins include the tetraspanin EV markers CD9, CD63, and CD81, which have each been identified as highly enriched in various EV subpopulations compared to their respective levels in cells.^[^ ^15^, ^16^, ^17^, ^18^ ^]^ In EV‐producing cells, CD9 and CD81 are generally localized to the plasma membrane while CD63 is localized to endosomal bodies, however the localization and expression levels of these proteins can be variable between cell types. This coincides with findings that CD9 and CD81 are generally more abundant in isolated EVs, suggesting that for certain cell types, ectosomes may be secreted in greater amounts than exosomes.^[^ ^11^ ^]^ Additionally, recent findings have shown that altering the C‐terminus of CD63 disrupts the endosome localization signal and causes the protein to translocate to the plasma membrane. When CD63 is localized to the plasma membrane, the number of CD63+ EVs produced increases by over sixfold.^[^ ^19^ ^]^ Our group has utilized CD9, CD63, and CD81 to load protein cargo into EVs for engineered EV production.^[^ ^20^, ^21^ ^]^ Although efficient EV cargo‐loading has been achieved, the impact of these molecular engineering approaches on EV biogenesis is not fully understood.

Exosome biogenesis is a complex network of several interdependent pathways, but can be broadly classified as Endosomal Sorting Complexes Required for Transport (ESCRT)‐dependent or ESCRT‐independent.^[^ ^22^ ^]^ ESCRT‐dependent pathways involve the formation of the ALIX/Syntenin/Syndecan tripartite to trigger ILV formation.^[^ ^23^ ^]^ ESCRT‐independent pathways of exosome production mainly involve ceramide, a lipid that is a product of neutral sphingomyelinase (SMPD2 and SMPD3) activity.^[^ ^24^ ^]^ Ceramide is involved in several cellular processes, including the organization of the ILV membrane and therefore the ESCRT‐independent formation of exosomes in the MVBs.^[^ ^25^, ^26^ ^]^ Recently, ceramide has also been SMPD3 with the small molecule GW4869 or siRNA has been shown to decrease exosome production while increasing ectosome production from the plasma membrane.^[^ ^27^ ^]^

Increasing EV production has become an important focus in the EV field to promote the potential of EVs as therapeutic agents from a production point of view. Various approaches have been developed for increasing EV output in cell culture.^[^ ^28^ ^]^ In this regard, an essential production factor to optimize is the culture media. The major media component which must be considered in EV production is serum, as media sera such as fetal bovine serum (FBS) introduce several factors which may interfere with endogenous EV activity and production such as ribonucleic artifacts and exogenous microparticles.^[^ ^29^, ^30^ ^]^ A major concern is the presence of exogenous serum EVs which may be biologically active and can interfere with the EV biogenesis, isolation, and downstream analysis of the purified EVs.^[^ ^31^ ^]^ Commercially available EV‐depleted serum exists but manufacturers are generally not transparent with the methods used to deplete EVs from the serum and these sera may have reduced capacity to support cell growth.^[^ ^32^ ^]^ Furthermore, use of FBS in culturing conditions for the production of EVs for therapeutic applications may not be ideal due to its animal origin. Therefore, identifying new serum‐free culture conditions for EVs production will greatly benefit the large‐scale production of therapeutic EVs.

Our group has previously shown that serum‐free media conditions can greatly increase the quantity of EVs produced without altering the size or physical properties of the EVs.^[^ ^33^ ^]^ These findings essentially make the complications which arise when using serum in EV‐production media obsolete. Recent research in the field has focused on identifying the signaling factors which prompt cells to increase EV production while maintaining cell viability in the absence of serum. Exogenous epidermal growth factor (EGF) has been found to significantly decrease EV production.^[^ ^34^ ^]^ Conversely, addition of exogenous heparanase to culture media stimulates EV production and alters protein composition of EVs.^[^ ^35^ ^]^ However, investigations into different cell culture media conditions do not always give straightforward insights. For example, both media containing high glucose levels and media subjected to glucose depletion have been found to stimulate EV production and alter EV bioactivity.^[^ ^36^, ^37^, ^38^ ^]^ Additionally, a wide range of small molecule compounds have been observed to alter EV production levels.^[^ ^39^ ^]^

In this study, we aim to discern the effects of different media conditions on the production of engineered EVs. The use of conventional EV quantification method for quantifying EV concentrations from unpurified cell culture conditioned medium can often be misleading due to high background associated with protein aggregates and FBS EVs. Therefore, to evaluate EVs secretion in an unbiased and a semi‐high throughput manner, we have utilized a previously described EV engineering approach which allow us to load a thermostable luciferase (TLuc) reporter protein exclusively into EVs.^[^ ^20^ ^]^ Hence, providing a mean of direct EV quantitation in conditioned media from cell culture (CM) without any rigorous purification method. We then investigate the effects of various media components, including exogenous EVs, on EV production in regards to EV quantity and EV composition. Furthermore, we employ RNA sequencing to elucidate transcriptional changes in EV production pathways which result from the tested media conditions. Finally, we use pharmacological‐ and gene‐interference approaches to confirm the role of the identified pathways.

## 2 Results

### 2.1 Culturing HEK293T Cells in Opti‐MEM Enhances EV Secretion

To assess the effect of different culturing conditions on EV production, we chose to utilize an EV engineering approach which loads an exogenous reporter protein into EVs. When using any expression construct‐based engineering approach, various factors such as promoter activity in different cell environments and transient transfection can significantly affect the outcome of the experiments. Therefore, we sought to address these concerns by generating HEK293T cell lines, which were transduced to stably express a tetraspanin (CD9, CD63, or CD81)‐TLuc fusion protein as described previously.^[^ ^20^ ^]^ Furthermore, to minimize the variation associated with promoter activity upstream of the transgene in various culturing conditions, we utilized a strong constitutive promoter EF1A which has previously been shown to have a high and stable promoter activity. The cells were referred to herein as HEK CD9‐TLuc, HEK CD63‐TLuc, and HEK CD81‐TLuc, respectively, and unedited HEK293T cells are referred to as HEK wt. Previous work has demonstrated that the optimal timeline for EV production and harvesting is as follows: cells are seeded in serum‐containing growth media until they are ≈70% confluent, and then the culture media is changed to an EV‐production media which should not contain exogenous EVs. This EV‐production media is then collected after 48 h, cellular debris is removed, and EVs can be quantified as a means of luciferase activity.

**Figure 1:**
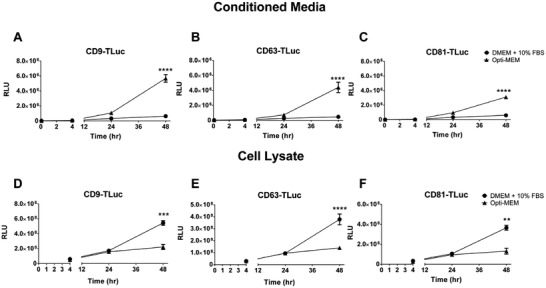
Opti‐MEM increases EV production in TLuc‐engineered cell lines over DMEM + 10% FBS. A–C) Luciferase activity measured from conditioned Opti‐MEM or DMEM + 10% collected from A) CD9‐TLuc, B) CD63‐Tluc, or C) CD81‐Tluc EV producing cells at 5 min, 4, 24, and 48 h. D–F) Luciferase activity measured from cell lysate of D) CD9‐Tluc, E) CD63‐Tluc, or F) CD81‐Tluc EV‐producing cells cultured in Opti‐MEM or DMEM + 10% FBS at 4, 24, and 48 h. Luciferase activity is reported as RLU. For all experiments, the error bars show the standard deviation of three biological replicate measurements (mean ± SD, *n* = 3). Statistical significance (*p < 0.05, **p < 0.01, ***p < 0.001, and ****p < 0.0001) was calculated with two‐way ANOVA test with Bonferroni correction for multiple comparisons. Intra‐experimental variability can contribute to discrepancies in RLUs between experiments.

The first aim of this study was to compare the EV production profiles between the cell lines utilizing the different tetraspanin‐luciferase fusions. Conditioned medium (CM) from different engineered cell lines displayed similar patterns of EV production (**Figure** **1**a–c). In all cases, and regardless of the tetraspanin construct used, significantly higher levels of luciferase signals in the CM were observed upon using Opti‐MEM as production media compared to DMEM + 10% FBS. The luciferase levels reflect the number of engineered EVs produced, suggesting higher amounts of engineered EVs are produced in Opti‐MEM. Across the timepoints tested, the highest levels of luciferase in Opti‐MEM were observed at 48 h. Thus, 48 h was set as the suitable timepoint for collection and analysis in later experiments.

We then sought to confirm that the observed enhancement in luciferase signal correlates with nanoparticle concentration in the CM. Nanoparticle tracking analysis (NTA) patterns observed from CD63‐TLuc CM corroborate the EV signal measured in DMEM + 10% FBS and in Opti‐MEM. NTA particle concentrations were plotted against the luciferase values from the same Media (Figure S1a, Supporting Information). Conditioned Opti‐MEM contained the highest concentrations of particles and the highest luciferase values. Correspondingly, DMEM + 10% FBS gave lower luciferase signals and had a lower particle concentration, although these were not expected to correlate due to the presence of exogenous serum EVs in the FBS. To illustrate the overall distribution of particles size and concentration, curves are shown for the total particles in the various conditioned media (Figure S1b, Supporting Information). Additionally, we wanted to exclude the possibility of a false‐positive EV signal, which could arise from luciferase protein which was not vesicle‐associated. Previous work has observed that some luciferase variants such as nanoluciferase can be expressed as soluble protein and secreted freely into the cell media, even when expressed as a conjugate to an EV marker protein.^[^ ^20^ ^]^ To ensure this was not the case with the TLuc constructs, we subjected the Opti‐MEM CM from all tetraspanin cell lines to treatment either with Triton X‐100, Proteinase K, or both (Figure S2, Supporting Information). The rationale behind this is that if luciferase was present in the CM outside of EVs, proteinase K would diminish the luciferase signal. However, if the luciferase was present only in EV lumen, it would be protected from proteinase K and proteinase K would only diminish the luciferase signal when EVs were first treated with Triton X‐100. The results indicate that the luciferase signal is strong only when Triton X‐100 is used to disrupt the vesicle membranes, and no Proteinase K is present, confirming that the luciferase is present in the EV lumen.

Interestingly, the higher levels of luciferase in Opti‐MEM CM coincide with depleted luciferase levels in the Opti‐MEM cultured cell lysate, as compared to cells cultured in DMEM + 10% FBS, which retained higher levels of luciferase intracellularly (Figure 1d–f). These findings imply that the increased luciferase levels in Opti‐MEM CM are not simply the result of increased construct expression in the EV‐producing cells. The increased luciferase signal observed in the lysate of the DMEM + 10% FBS cultured cells could potentially be due to certain components of the cell media impairing EV biogenesis and subsequently leading to construct accumulation in the cells. To investigate this further, we proceeded to analyze the component reagents in cell culture media for their effect on the production of luciferase‐loaded EVs.

### 2.2 Serum is a Key Inhibiting Component of Culture Media in the Production of Engineered EVs

Our investigation began with the most obvious variable component between the above‐mentioned media conditions: FBS. Serum can influence cell viability and proliferation through a plethora of signaling pathways. To exclude the possibility that serum was influencing EV production by impacting cell viability, HEK wt cells were cultured for EV production in Opti‐MEM or DMEM + 10% FBS, and then cell viability was assessed in each of the media conditions (Figure S3, Supporting Information). No significant decrease in viability was observed.

**Figure 2:**
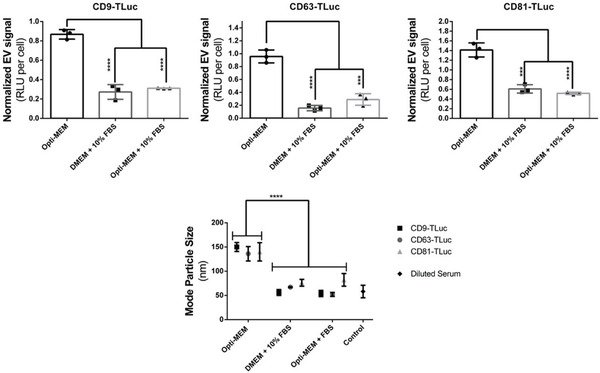
Culture media with serum inhibits EV production. A–C) Luciferase activity measured from conditioned Opti‐MEM, DMEM + 10% FBS, or Opti‐MEM + 10% FBS collected from A) CD9‐Tluc, B) CD63‐Tluc, or C) CD81‐Tluc EV producing cells at 48 h. Luciferase values, reported as RLU, were normalized to the cell number as measured by DAPI staining and flow cytometry. D) Mode size of nanoparticles as reported by NTA in conditioned media from CD9‐TLuc, CD63‐TLuc, or CD81‐TLuc cells cultured in Opti‐MEM, DMEM + 10% FBS, or Opti‐MEM + 10% FBS. Luciferase activity is reported as RLU normalized to total cell count from each biological replicate. For all experiments, the error bars show the standard deviation of three biological replicate measurements (mean ± SD, *n* = 3). Statistical significance (*p < 0.05, **p < 0.01, ***p < 0.001, and ****p < 0.0001) between experimental groups was calculated with one or two‐way ANOVA with Bonferroni correction for multiple comparisons. Intra‐experimental variability can contribute to discrepancies in RLUs between experiments.

To then explore the impact of serum on EV production in relation to cell proliferation, the experiments from Figure 1 were again performed, this time with the additional condition of Opti‐MEM supplemented with 10% FBS (Opti‐MEM + 10% FBS). Additionally, cells from each well were counted and this time the luciferase signal from the corresponding CM was normalized to cell count (**Figure** **2**a–c). After 48 h, Opti‐MEM CM contained the highest EV signal per cell, corroborating the results in Figure 1. Additionally, the results were similar across the different tetraspanin constructs, again supporting that the increase in EV signal is dependent on the cell media composition rather than the expression of the EV‐engineering construct. Interestingly, the presence of serum in Opti‐MEM drastically decreased the EV signal to comparable levels with DMEM + 10% FBS, implying that serum has an inhibitory effect on EV production.

We then proceeded to characterize the size profiles of the total particles in the various CM using NTA (Figure 2d). The mode particle sizes were plotted and compared against a control containing phosphate‐buffered saline (PBS) supplemented with 10% FBS (diluted serum). The findings indicate that particles in Opti‐MEM CM display a larger mode size, between 130 and 160 nm, for all cell lines. This size range is characteristic of EVs such as exosomes and ectosomes.^[^ ^40^ ^]^ However, in the media which contain serum, the mode particle size was much smaller, varying between 50 and 90 nm. This particle size suggests that there are small nanoparticulate species in FBS which are present in higher numbers than the EVs, possibly including protein aggregates, low‐density lipoprotein particles, and others.^[^ ^41^ ^]^ The findings here indicate that the nanoparticle profile of Opti‐MEM CM is heavily determined by the presence of the produced EVs, while serum‐containing media unsurprisingly contain other nanoparticle species which could potentially be responsible for inhibiting EV biogenesis. As serum had a strikingly potent ability to inhibit EV production, we next investigated whether EV production could be restored by removal of any individual components of serum.

### 2.3 Individual Serum Components Exert Differential Effects on EV Production

Having identified serum as a major influencing component of cell culture conditions, we sought to determine which components in serum have an inhibitory effect on EV production. While it is out of this project's scope to analyze all the components of serum individually, we selected components that we predicted would have an inhibitory nature on EV production by directly impacting the exocytic pathways. The components that we selected to test included albumin, globulin, fibronectin, and exogenous EVs.

**Figure 3:**
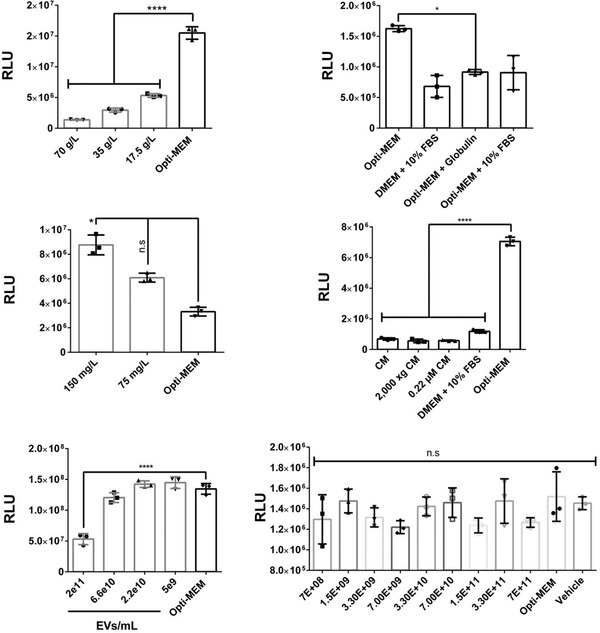
Media components differentially influence CD63‐TLuc EV production. A) Luciferase activity measured from conditioned Opti‐MEM supplemented with albumin. B) Luciferase activity measured from conditioned media, including Opti‐MEM with supplemented globulin. C) Luciferase activity measured from conditioned Opti‐MEM supplemented with fibronectin. D) Luciferase activity measured from CM collected from various EV‐production media conditions. CM was collected from HEK wt cells after 48 h and cell debris was either not removed (CM) or removed by centrifugation (2000 × *g*) or by centrifugation and filtration (0.22 × 10^−6^ m). These CMs were then given as production media to CD63‐Tluc cells for 48 h, and then luciferase levels were measured and compared to Opti‐MEM and DMEM + 10% FBS CMs. E) Luciferase activity measured from Opti‐MEM CM which had been supplemented with various numbers of HEK wt EVs. F) Luciferase activity measured from conditioned Opti‐MEM supplemented with various numbers of MC3‐LNPs. All measurements of conditioned media were taken at 48 h after media change. Luciferase activity is reported as RLU. Statistical significance (*p < 0.05, **p < 0.01, ***p < 0.001, and ****p < 0.0001) was calculated with one‐way ANOVA test with Bonferroni correction for multiple comparisons. For all experiments, error bars show the standard deviation of three biological replicate measurements (mean ± SD, *n* = 3). Intra‐experimental variability can contribute to discrepancies in RLUs between experiments.

Albumin and globulin are two high molecular‐weight protein species in FBS that have a propensity for aggregation in cell culture. We sought to test the effects of albumin by diluting albumin into Opti‐MEM to various final concentrations for production media (**Figure** **3**a). Indeed, increasing concentrations of albumin led to decreased levels of CM luciferase signal, as did the addition of globulin into Opti‐MEM which led to a reduction in the CM luciferase signal comparable to the levels that of Opti‐MEM + 10% FBS (Figure 3b). The findings support the notion that large serum proteins which are known to form nanoparticle‐like aggregates can inhibit production of the engineered EVs. This led us to ask if this effect could be seen from other proteins which are known to interact with EVs. Fibronectin has been implicated as an EV surface‐bound signaling moiety that can increase EV uptake in recipient cells.^[^ ^42^ ^]^ We expected that exogenous fibronectin may therefore influence the release of EVs during EV production. Interestingly, when added to Opti‐MEM, fibronectin was found to increase EV production (Figure 3c). This finding seemingly excludes the possibility that fibronectin is an inhibitory component of serum, and further implicates it as a potential booster of EV production when used in serum‐free media. However, it is important to note that the fibronectin we added to Opti‐MEM was isolated, and thus was not pre‐associated with exogenous EVs, as would be the case in FBS. Therefore, the relevance of this finding to EV‐containing samples should be separately investigated.

We were particularly interested to investigate the effects that exogenous EVs could have on the EV‐producing cells. Exogenous EVs could, e.g., inhibit the release of newly produced EVs from cells in a competitive manner, which would partially explain the decreased EV signal in serum‐containing CMs. Conversely, exogenous EVs could inhibit EV production through the presence of an active EV‐associated protein such as fibronectin. To address this, we collected Opti‐MEM CM from HEK wt cells, centrifuged and filtered it to remove cell debris, and then used this CM as production media for CD63‐TLuc EV production (Figure 3d). The rationale behind this experiment is that the wild‐type CM contains HEK wt EVs, among other factors, that may inhibit the production of the CD63‐TLuc EVs. Indeed, compared to fresh Opti‐MEM, the conditioned Opti‐MEM led to a decrease in the number of EVs produced by over 70%, a similar level as fresh DMEM + 10% FBS. The results seemingly confirm that exogenous cell media components such as EVs could impact EV production.

To further probe the effect of exogenous EVs, we sought to directly supplement isolated exogenous EVs into the EV production media. HEK wt EVs were produced and isolated in PBS as described previously by our group.^[^ ^40^ ^]^ We then diluted these EVs to various concentrations in Opti‐MEM which were then used as production media for CD63‐TLuc EVs (Figure 3e). Importantly, we found that above a certain concentration (2.2e10 EVs per mL), the levels of produced luciferase‐EVs steadily declined. These findings indicate that exogenous EVs are capable of decreasing the production of new EVs.

Since we were able to demonstrate that exogenous EVs could inhibit engineered EV production, we looked to understand if this holds true for other nanoparticulate species. Both exogenous EVs and the abovementioned protein aggregates inhibit EV production by either influencing specific signaling events or as a result of their general behavior as nanoparticles. To explore this further, we chose to utilize lipid nanoparticles (LNPs) as an additional nanoparticulate species to be supplemented in Opti‐MEM. Following the same treatment timeline that we used for EVs, we treated CD63‐TLuc cells with DLin‐MC3‐DMA lipid nanoparticles (MC3‐LNPs) at a broad range of concentrations (Figure 3f). MC3‐LNPs are well‐characterized and have a size profile resembling that of small EVs (generally 40–120 nm, depending on formulation^[^ ^43^ ^]^). Interestingly, there was no significant effect on the quantity of produced EVs. The results imply that the inhibitory nature of albumin, globulin, and exogenous EVs are not broadly attributed to all nanoparticles but are rather specific to those macromolecular/nanoparticulate species.

Having identified that the serum components albumin and exogenous EVs reduce the production of TLuc EVs, we attempted to bolster the production of TLuc EVs in Opti‐MEM by adding EV‐depleted serum and albumin‐depleted serum (Figure S4, Supporting Information). The use of Opti‐MEM + 10% EV(‐) FBS production media did not increase the TLuc signal over the Opti‐MEM + 10% FBS production media. Further, EV production could not be significantly increased with albumin‐depleted serum nor serum which had been depleted of both albumin and EVs. The findings imply that serum‐free Opti‐MEM is the optimal cell culture media for EV production, and the deleterious effects of serum could not be overcome by removing those identified components.

### 2.4 Other Culture Media and Media Components are not as Effective as Opti‐MEM at Bolstering EV Production

Having demonstrated the negative effect of FBS on EV production, we aimed to test other serum‐free and low‐serum media for their ability to increase EV production. These included FreeStyle expression media, a protein‐free, animal source‐free media which supports cell transfection and growth and has been used for antibody production;^[^ ^44^ ^]^ minimal essential media (MEM), one of the most commonly used, established cell culture media; and virus production serum‐free media (VP‐SFM), a serum‐free, ultra‐low protein media optimized for virus production. Cells were cultured for EV production and the CMs were analyzed as described above, however none of these media were able to enhance EV production in a manner similar to Opti‐MEM (Figure S5, Supporting Information). As it became increasingly evident that Opti‐MEM is the best‐formulated media for engineered EV production, we next sought to explore potential signaling factors which could be supplemented into Opti‐MEM to increase EV production.

To this end, signaling factors were selected which promote cell growth and viability such as insulin, transferrin, selenium, and L‐glutamine. Commercially available insulin, transferrin, selenium (ITS) solution was added to the Opti‐MEM for 48 h but did not yield increased EV production (Figure S6a, Supporting Information). Continuing along this experimental line, we also tested Opti‐MEM supplemented with various concentrations of GlutaMAX, a commercially available dipeptide L‐alanine‐L‐glutamine (Figure S6b, Supporting Information). The absence of glutamine has been shown to alter cancer exosome biogenesis, and we were therefore curious to examine whether the addition of GlutaMAX would impact EV production.^[^ ^45^ ^]^ However, the addition of GlutaMax did not induce any significant changes in Luciferase‐EV production.

After exploring these various media and media components, we proceeded to investigate the other factors involved in production of engineered EVs. It was of utmost importance to determine whether Opti‐MEM's ability to bolster EV production was related to the expression of the engineering constructs.

### 2.5 Engineering Construct Expression Causes Translocation of EV Markers CD9, CD63, and CD81

Up to this point, we had primarily used quantitative methods to determine the levels of EV‐production. We therefore sought to determine the underlying cellular changes that influence EV production. The use of the tetraspanin proteins CD9, CD63, and CD81 in our EV‐loading constructs could alter the biogenesis routes and the compositions of the produced EVs. To this end, we sought to characterize the behavior of the tetraspanin constructs in the EV‐producing cells in regards to cellular localization.

**Figure 4:**
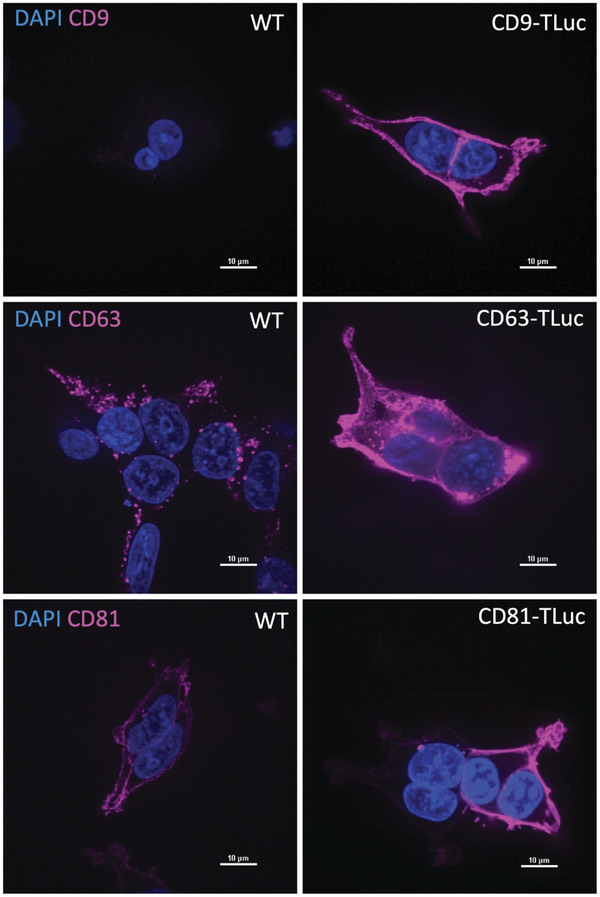
Tetraspanin localization in HEK wt and engineered cell lines. A) Fluorescence microscopy visualizing the localization of CD9, CD63, and CD81 in wildtype HEK293T cells and in their respective engineered EV‐producing cells in DMEM + 10% FBS. Nuclei (blue) are shown to identify cells. Immunofluorescence staining for CD9, CD63, and CD81 (pink) identifies the localizations of the respective EV‐related tetraspanin proteins. Scale bars shown for size.

As EV subpopulations can be distinguished based on the cellular localization of their biogenesis, we employed a microscopic approach to visualize the constructs. Ectosomes originate from the plasma membrane while exosomes originate from endocytic bodies within the cell. To better understand the intracellular localizations from which the engineered EVs were originating, we cultured cells as described for EV production, using Opti‐MEM as the EV production media, and then performed immunofluorescence staining for each of the tetraspanin EV markers in both HEK293T wt cells and in each of the stable cell lines expressing the respective tetraspanin construct. The localizations of the EV‐producing constructs were then visualized via fluorescence microscopy (**Figure** **4**).

All tetraspanin constructs were present in the plasma membranes of the engineered cells. CD9, which colocalizes to the plasma membrane in low quantity in HEK wt cells (undetectable in Figure 3a), was heavily enriched in the plasma membrane of the CD9‐TLuc stable cells. The same was found for CD63 which is exclusively endosome‐associated in HEK wt cells. In CD63‐TLuc cells, endosomal punctate were still visible, however CD63 was additionally present in the plasma membrane. CD81 is typically localized to the plasma membrane in HEK wt cells, and in the CD81‐TLuc cells, the signal coming from the plasma membrane was much brighter, suggesting an enrichment in membrane‐associated CD81. It is important to note that the expression of the constructs was not homogenous between individual cells, as can be seen in the image of CD81‐TLuc cells (two cells appear with strong/bright membrane signal, and two cells appear with weaker membrane signal).

Further, we investigated whether the localizations of these tetraspanins were in some way dependent on cell culture conditions. Each of the engineered cell lines were cultured as described for EV production, in either DMEM + 10% FBS or Opti‐MEM for 48 h. Immunofluorescent staining was again performed to visualize the tetraspanins. Figure S7 in the Supporting Information shows that there are no obvious visual differences in the tetraspanin localizations between the cells cultured in Opti‐MEM and the cells cultured in DMEM + 10% FBS. The qualitative results of these microscopy experiments imply that the localizations of the tetraspanin proteins CD9, CD63, and CD81 are impacted to a larger extent by the overexpression of our engineering constructs than the cell culture conditions.

### 2.6 Validation of TLuc Engineered‐EV Production

As the microscopy results demonstrated, the engineering construct had a considerable impact on the endogenous behavior of the tetraspanins. We believed it crucial to our study to ensure that the increased EV production observed in Opti‐MEM was not simply the result of differential construct expression in the EV‐producing cells. To this end, we sought to replicate the increased EV production in wild‐type cells. We collected CM from HEK wt and wild‐type mesenchymal stem cells (MSC wt) that had been cultured in either Opti‐MEM or DMEM supplemented with 10% EV‐depleted FBS (DMEM + 10% EV(‐) serum) for EV production (Figure S8, Supporting Information). EVs from MSCs have been reported to have anti‐inflammatory and regenerative potentials similar to those of MSCs, and MSCs are widely viewed as a promising cell source for clinically relevant therapeutic EVs.^[^ ^7^, ^46^ ^]^ The rationale behind using EV‐depleted serum for this experiment was to ensure that exogenous EVs were not contributing to the particle count in the serum‐containing samples. Additionally, to reduce variability between MSC and HEK proliferation rates, nanoparticle concentrations were normalized to cell counts. For both HEK wt and MSC wt cells, Opti‐MEM led to significantly greater particle concentrations without compromising the cell viability, corroborating the patterns of EV production observed from the TLuc‐engineered cell lines. Additionally, the results support the use of Opti‐MEM as an EV‐production media for therapeutically relevant cell sources.

Importantly, we employed several minimal information for studies of extracellular vesicles (MISEV)‐compliant EV‐characterization techniques to confirm that our EV quantitation approach was indeed measuring EVs.^[^ ^30^ ^]^ Electron microscopy (EM) was performed on CM produced from HEK wt cells cultured as described for our experiments. The CM harvested from these cells was further subjected to MISEV‐compliant EV‐isolation techniques, namely, filtration and ultracentrifugation. Characteristic EV morphology can be observed in several of the visualized particles (Figure S9, Supporting Information).

We then sought to characterize the protein composition of EVs to determine whether EV composition was impacted by the cell culture conditions. First, western blot was performed for the known EV markers Tsg101, Syntenin, and CD81 (Figure S10, Supporting Information), confirming the presence of these EV markers in EVs isolated from Opti‐MEM CM. Interestingly, CD81 was not present in detectable quantity in the DMEM + 10% FBS CM. In addition to western blot, EV surface protein composition was analyzed with a multiplexed, bead‐based flow cytometry assay (MACSPlex) as described previously,^[^ ^47^ ^]^ we probed for the presence of 37 different EV markers simultaneously without the need for a CM‐processing step, allowing us to detect, quantify, and compare EV surface compositions between the different production CMs. Various HEK wt CMs including DMEM + 10% FBS, Opti‐MEM, Opti‐MEM + 10% FBS, and DMEM + 10% EV‐depleted FBS were collected after 48 h and analyzed with the MACSPlex assay (Figure S11, Supporting Information). Importantly, the multiplexing function of this analysis ensures that only vesicle‐associated protein is quantitated. For our purpose, the quantitation of vesicular CD9, CD63, and CD81 are highlighted so that the ratios of these markers could be compared between the media conditions tested. We detected generally higher levels of EV surface marker signals when analyzing Opti‐MEM, especially for lower abundant EV markers, consistent with the observation that more EVs are present in Opti‐MEM. For the more abundant EV markers such as the tetraspanins CD9, CD63, and CD81, we observed higher absolute signals but no overall change in their patterns across the CMs. Indeed, the ratios of the tetraspanins to each other in the HEK wt cells closely mirrored both the NTA profiles of the HEK wt CM and the ratios of luciferase activity seen in the construct‐expressing cells, even between CM conditions. In conclusion, these results confirm that Opti‐MEM CM contained the most HEK wt EVs, which agrees with the findings from the engineered cell lines in Figure 1. Additionally, DMEM + 10% FBS contained the least amount of EVs, further supporting the results from the engineered EV experiments. Taken together, these results indicate that the overall tetraspanin composition of released EVs is not significantly different between the media conditions, which in turn implies that the increased EV production observed in Opti‐MEM cannot be assigned to a certain subset of EVs, but to a general increase of released EV quantities.

Importantly, the above results corroborate the effects of Opti‐MEM on EV production between the engineered EVs and wild‐type EVs. The findings imply that although our constructs lead to a loss of endogenous tetraspanin activity in the EV‐producing cells, EV production depends more on the culture media composition.

### 2.7 Media Serum Induces Transcriptional Changes in EV‐Producing Cells

Having demonstrated that specific media components can affect EV production while others do not, we sought to further investigate whether these changes occurred on a general cellular response level (e.g., the cell maintaining a nanoparticulate equilibrium with its environment), or if the changes were the result of specific alterations to intracellular pathways. One explanation could be that Opti‐MEM impacts EV production on a transcriptional level. To explore this, we first aimed to determine the effects on EV production by replacing Opti‐MEM after 24 h, compared to the normal 48 h treatment. We collected CD63‐TLuc Opti‐MEM CM after 24 h, replaced the media with fresh Opti‐MEM, and then again collected the CM after another 24 h period. The rationale behind this is that by replacing the Opti‐MEM after the first 24 h, the extracellular environment would essentially be depleted of EVs and proteins while simultaneously replenished with the signaling factors in Opti‐MEM. If there was no impact on cumulative EV production during the total 48 h, it could indicate that EV production was determined on a transcriptional level. We compared luciferase signals from these separate 24 h windows to the luciferase quantities of CM collected following our normal production period of 48 h (Figure S12, Supporting Information). Importantly, we observed that the sum of the signals from CM acquired after the first 24 h and the second 24 h periods was equal to the total signal after a single 48 h period, indicating that the EV production may be a result of transcriptional changes.

**Figure 5:**
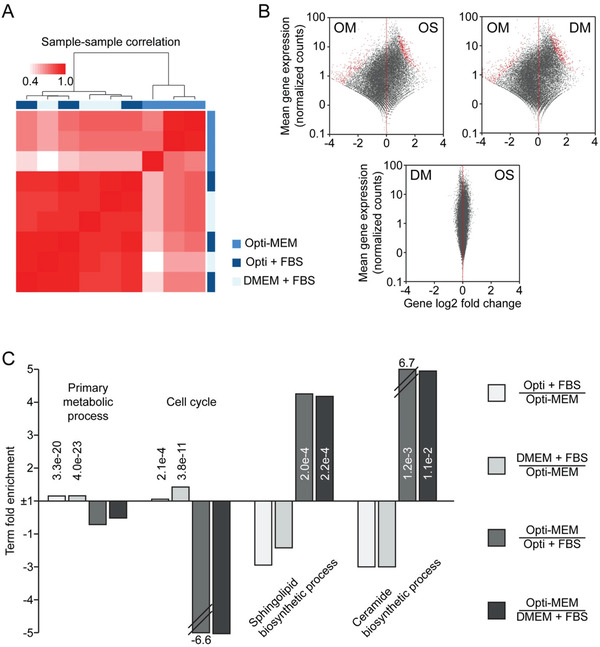
RNA sequencing of HEK wt EV‐producing cells in different EV‐production media. A) Heatmap of sample to sample correlations organized by hierarchical clustering of triplicate samples. Each condition was analyzed with *n* = 3. B) Deseq2 scatterplot of gene's fold changes and mean count levels. Differentially expressed genes with adjusted *p*‐value < 0.001 are shown in red. C) Gene ontology term fold enrichment of genes significantly upregulated in the comparisons displayed over all differentially expressed genes. *p*‐Values for each set of differentially expressed genes are inset if <0.05. Abbreviations: OM; Opti‐MEM, OS; Opti‐MEM supplemented with 10% FBS, DM; DMEM supplemented with 10% FBS.

We then sought to investigate which cellular processes were being impacted. To this end, HEK wt cells were cultured in serum‐free Opti‐MEM, Opti‐MEM + 10% FBS, or DMEM + 10% FBS, and then RNA was isolated from the cells and sequenced. Hierarchical clustering revealed that the transcriptional profiles of cells grown in Opti‐MEM correlated least with those grown in the media‐containing serum (**Figure** **5**a). The transcriptional profiles of cells grown in DMEM + 10% FBS clustered with those grown in Opti‐MEM‐containing serum, suggesting that the transcriptional differences seen in the Opti‐MEM‐cultured cells were due to the absence of serum rather than the additional signaling factors in the Opti‐MEM. Furthermore, the fold changes observed in transcript levels revealed that extensive differential genes expression could be observed in comparisons between Opti‐MEM and both other conditions, but not between the serum‐containing conditions (Figure 5b).

The differentially expressed genes were then subjected to gene ontology analysis to determine which molecular and biological processes were affected by the media conditions (Figure 5c). The enrichment levels of genes comprising a certain gene ontology term were compared between the different media conditions in which cells were cultured for EV production. It can be reasonably expected that the presence/absence of serum would impact the basic functions of the cells to grow and divide, and this is confirmed by the downregulation of genes in serum‐containing media related to “primary metabolic process” and “cell cycle” when Opti‐MEM + 10% FBS and DMEM + 10% FBS are compared to Opti‐MEM. A list of the top ten gene ontology hits for each media comparison is included in Table S1 in the Supporting Information. Of interest to our study was the fold enrichment in the GO terms related to EV production pathways, particularly “sphingolipid biosynthetic process” and “ceramide biosynthetic process” observed in Opti‐MEM. The sphingolipid and ceramide pathways are key pathways which influence exosome production.^[^ ^48^ ^]^ Both of these GO terms were upregulated in the Opti‐MEM samples compared to the serum‐containing samples, indicating that cells cultured in Opti‐MEM have increased levels of sphingolipid and ceramide synthesis on a transcriptional level. Taken together, these results imply that the ceramide‐dependent exosome biogenesis pathway is responsible, at least in part, for the increase in engineered EVs which are produced in Opti‐MEM.

### 2.8 Inhibition of SMPD2 Exerts Differential Effects on Serum‐Containing and Serum‐Free Media

**Figure 6:**
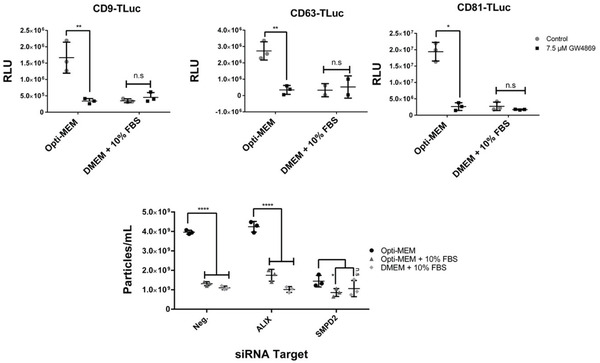
Ceramide pathway inhibition in CD63‐TLuc EV producing cells. A–C) Luciferase activity measured from conditioned Opti‐MEM or DMEM + 10% FBS containing A) CD9‐Tluc, B) CD63‐Tluc, or C) CD81‐Tluc cells supplemented with 7.5 × 10^−6^ m GW4869. D) Nanoparticle tracking analysis performed on CM from cells treated with siRNAs targeting EV production pathways. Luciferase values are reported as RLU. Statistical significance (*p < 0.05, **p < 0.01, ***p < 0.001, and ****p < 0.0001) was calculated with one‐way or two‐way ANOVA test with Bonferroni correction for multiple comparisons. For all experiments, the error bars show the standard deviation of three biological replicate measurements (mean ± SD, *n* = 3). Intra‐experimental variability can contribute to discrepancies in RLUs between experiments.

Having identified sphingomyelin‐related pathways as altered in Opti‐MEM‐cultured cells, we next sought to confirm to what extent these transcriptional changes were impacting EV production levels. To first explore this, cells were cultured for EV production in Opti‐MEM supplemented with GW4869, a well‐cited neutral sphingomyelinase inhibitor capable of decreasing exosome production.^[^ ^49^ ^]^ For each of the engineered cell lines, GW4869 significantly decreased engineered‐EV production in Opti‐MEM (**Figure** **6**a–c). The EV signals in the DMEM + 10% FBS samples were not significantly different between the GW4869‐treated and ‐untreated groups. These findings further implicate the ceramide‐dependent EV production pathway as a primary route for increased EV production in Opti‐MEM.

Finally, we sought to determine whether all EVs, including endogenous EVs which did not contain the luciferase construct, were produced in Opti‐MEM in a manner dependent on ceramide activity. Cells were treated with siRNA targeting either ALIX, a key protein in ESCRTIII‐dependent, ceramide‐independent EV biogenesis, or siRNA targeting SMPD2, which directly interacts with ceramide during EV biogenesis. siRNA knockdown efficiencies were validated via reverse transcription polymerase chain reaction (RT‐PCR; Figure S13, Supporting Information). After siRNA treatment, NTA was performed to quantitate all EVs present in CM, not just the luciferase‐loaded EVs (Figure 6d). In agreement with our earlier findings, there were ≈50% fewer particles present in the serum‐free Opti‐MEM CM after treatment with the SMPD2 siRNA. The control siRNA targeting ALIX did not impart a significant decrease on total particle count in serum‐free Opti‐MEM. These results further support the role of the ceramide‐dependent exosome biogenesis pathway as a key contributing mechanism to the increased EV production which Opti‐MEM induces.

## 3 Discussion

Several studies have been conducted to date with the intent of optimizing the production of engineered EVs for both therapeutic and research purposes. The therapeutic application of EVs can include their use as a stand‐alone therapeutic delivery method (i.e., intravenous administration of EVs) or as part of a combinatorial strategy (i.e., EV‐embedded implantable biomaterials).^[^ ^50^ ^]^ Both of these approaches require highly optimized EV production. To date, much effort has focused on technical optimization, including improving purification techniques and increasing the absolute quantity of EVs which can be produced relative to the quantity of cells and reagents used.^[^ ^20^, ^21^, ^40^ ^]^ This has resulted in the establishment of a robust pipeline for producing EVs with relatively well‐characterized physical properties. However, the complexity and heterogeneity of EV biogenesis have obscured the changes which occur on a cellular level. In this study, we aim to discern and characterize the endogenous biological pathways at play and the extent to which these pathways are influenced through the EV‐engineering approaches.

Our group has previously reported that EVs produced in serum‐free Opti‐MEM culture media were greater in quantity than EVs produced in DMEM‐containing serum.^[^ ^33^ ^]^ This study formed a basis for our understanding of the impacts different media can have on EV production but it did not assess individual media components nor did it investigate underlying biological changes in the EV‐producing cells.

The first goal of this study was to establish our luciferase‐based EV quantitation method as a reliable reporter of EV production levels. Using engineered cell lines for EV quantitation inherently requires us to address the possibility that the expression of the engineering construct is imparting an effect on EV production. In this case, our construct is driven by a strong constitutive promoter to reduce variability between media conditions. However, the high levels of construct expression inevitably alter the localization of the tetraspanins in the EV‐producing cells as compared to wild‐type cells, confirmed in Figure 4. However, this does not impede our ability to use these constructs to investigate media‐dependent changes in EV production.

Further, we took particular care to correlate EV production from wild‐type cells (HEK and MSC) with the EV production signals from our luciferase‐engineered cell lines. Indeed, at 48 h, the EV‐production patterns observed from the wild‐type cells, as measured by NTA (Figure S8, Supporting Information) closely resembled the EV‐production patterns of the TLuc‐engineered cells, as measured by luciferase assay (Figure 1a–c).

It is important to note that our engineering approach has two shortcomings: first, we cannot claim that the endogenous functions of the tetraspanins used in our engineering constructs were conserved in the EV‐producing cells, and second, the construct expression was not homogenous across EV‐producing cells. However, we show that due to the heterogeneity of EV populations produced in cell culture, and by controlling our experiments with multiple EV characterization approaches, these were not necessarily detrimental for our purposes of quantitative EV characterization. The 2018 MISEV guidelines outline the necessity for reliable EV‐characterization tools. In this study, we combine the use of traditional techniques such as NTA, EM, western blot, and NTA with a recently developed, high‐resolution technique, namely, the multiplex bead‐based flow cytometry analysis. Taken together, these analyses show that our engineering approach reliably quantitates EV production. Additionally, the MACSPlex data indicate that the levels of the tetraspanins in wt‐EV populations were not significantly altered between the different production media (Figure S12, Supporting Information).

It is of course important to consider that small differences in cell viability can impart a strong effect on the number of nanoparticles produced by a given cell. For example, a cell undergoing apoptosis may release more particles than a healthy cell, with the majority of the apoptotic nanoparticles having undesirable characteristics. While Opti‐MEM did result in slightly lower cell viability (97% vs 98%, Figure S3, Supporting Information), we do not consider a 1% difference after 48 h of cell culture to be a driver of the significant increase in EV signal in Opti‐MEM. Additionally, we did not observe an increase in apoptosis‐related GO terms (Table S1, Supporting Information) when comparing Opti‐MEM to DMEM + 10% FBS.

By developing a reliable quantitation platform for luciferase‐loaded EVs, we were then able to characterize EV production across different media conditions. In cell culture, the differential presences of CD9, CD63, and CD81 across an EV population indicate that a heterogenous population of EVs is produced from several exocytic pathways. It was therefore unexpected when cells cultured in serum‐free Opti‐MEM produced significantly more engineered EVs regardless of the tetraspanin used in the engineering construct. This seemed to imply that Opti‐MEM is capable of enhancing the production of all EVs, regardless of their intracellular origin. Further, decreased levels of luciferase protein were retained in the cells grown in Opti‐MEM compared to cells grown in DMEM + 10% FBS or Opti‐MEM‐containing serum. Taken together, these findings imply that cells cultured in Opti‐MEM exhibit a different equilibrium threshold in terms of the quantities of CD9, CD63, and CD81 that are retained intracellularly versus the quantity which is packaged and subsequently exocytosed in EVs. Thus, the central focus of our study became determining whether this new equilibrium is the result of molecular signaling/interaction, physical competition, or transcriptional changes.

Our approach to determining a molecular interaction between media and the EV‐producing cells led our attempt to identify specific media components that increase EV production. We approached this by assessing other serum‐free media than Opti‐MEM, as well as testing the additions of various signaling factors to the production media. The different media we investigated, including VP‐SFM and FreeStyle expression media, and the media components such as GlutaMAX and ITS solution, did not enhance EV production over the levels seen with normal serum‐free Opti‐MEM. Additionally, it is an ongoing concern that commercial solutions of media additives may contain other particles and soluble factors which could contaminate the experiments, and for the production of EVs which have downstream applications, researchers must take care to avoid any such contaminants.

However, the addition of purified fibronectin protein to Opti‐MEM was able to induce an increase in EV production. The increase induced by fibronectin is of particular interest considering fibronectin has been associated with EV uptake.^[^ ^42^ ^]^ Therefore, we believe that there may exist macromolecular media components that exhibit biomolecular interaction with EV‐producing cells which are partially responsible for altering the levels of EVs produced.

Our findings also suggest the possibility of physical competition between some nanoparticulate species in cell media and the EVs being produced. This was first tested by adding albumin and globulin to Opti‐MEM production media. Both albumin and globulin are high‐molecular‐weight proteins which have propensity to aggregate, forming protein aggregates that can display properties similar to nanoparticles.^[^ ^51^ ^]^ The addition of albumin to cell media led to dose‐dependent decreases in luciferase EV production. Additionally, the presence of exogenous EVs in the production media also appeared to competitively inhibit the production of new EVs in a dose‐dependent manner.

However, these findings did not hold true with another nanoparticulate species, MC3‐LNPs. The fact that MC3‐LNPs did not inhibit EV production across a broad concentration range implies that the physical competition effect, if substantive, is specific only to certain protein species and exogenous EVs. Thus, we conclude that the inhibitory effect of DMEM + 10% FBS is not purely a result of physical competition. This was further supported by the use of both albumin‐depleted and EV‐depleted sera in EV production media which led to a partial recovery of EV production, but not close to the levels observed with serum‐free Opti‐MEM. At this point, we hypothesized that the decrease in EV production induced by serum‐containing media must be a result of the combined effects of certain molecular interactions and physical competition.

Next, we investigated transcriptional changes caused by serum‐free Opti‐MEM. The transcriptional differences observed between cells cultured in Opti‐MEM with and without serum were large, albeit perhaps unsurprising, as serum contains many signaling factors and proteins. However, our interest piqued at the observation that several exosome‐related genes were identified as significantly upregulated in cells grown in serum‐free Opti‐MEM. Further, Opti‐MEM + 10% FBS clustered with DMEM + 10% FBS in our hierarchical clustering model, implying that the absence of serum is the determining factor for these transcriptional changes rather than the additional signaling factors that Opti‐MEM is formulated with. This is not to say that Opti‐MEM's signaling factors are unimportant, however, as the ability to culture cells in the absence of serum, and to bolster EV production, is a function unique to Opti‐MEM.

The results from the GO analysis implicate ceramide and sphingolipid biogenesis as relevant affected processes. Ceramide is a central intermediate of sphingolipid metabolism and has been implicated in the formation of ILVs within the MVBs and is therefore necessary for the production of exosomes within the endocytic pathway. It stands to follow that the increase in the production of engineered EVs which we observe in Opti‐MEM is at least partially the result of significant upregulation in ceramide and sphingolipid metabolism.

To validate the GO analysis results, we treated cells in production media with GW4869, a known ceramide inhibitor. SMPD2 inhibition with GW4869 has been shown to decrease exosome production while increasing ectosome production in cancer cell lines. The significant decrease observed in Opti‐MEM CM further supports that a large portion of engineered EVs produced in serum‐free Opti‐MEM are produced via ceramide‐dependent ILV formation. Particularly of interest, cells cultured in serum‐containing production media showed no significant difference in EV production when treated with GW4869. Therefore, our findings regarding the role of ceramide‐dependent EV production may not pertain to other media conditions than serum‐free Opti‐MEM.

In conclusion, we demonstrate that certain serum components can decrease the production of engineered EVs. Both wild‐type cells and cell lines engineered for EV loading produce significantly more EVs when cultured in serum‐free Opti‐MEM conditions. In the engineered cell lines, the increase in EV production is concurrent with the translocation of the EV‐loading construct to the plasma membrane. This is significant, considering that the inhibition studies implicate the role of the exosome biogenesis pathway as a major contributing pathway to the release of engineered EVs. Hence, we hypothesize that serum‐free Opti‐MEM is encouraging EV release as a result of a combination of physical, molecular, and transcriptional effects. By disentangling EV biogenesis, optimized production methods can be developed, bringing EVs one step closer to reaching their potential as a next‐generation therapeutic.

## 4 Experimental Section

### Cell Lines

HEK293T cell lines expressing CD9‐Luc, CD63‐Luc, or CD81‐Luc were generated via stable transduction with constructs encoding ThermoLuc (TLuc) luciferase recombinantly fused to codon‐optimized CD9, CD63, or CD81, as previously described.^[^ ^20^ ^]^

### Cell Culture

Catalog numbers for all cell culture reagents are included herein in accordance with ISEV recommendations. HEK293T‐transduced cell lines were cultured in high glucose DMEM medium (Gibco, USA, catalog number: 10566016) supplemented with 10% FBS (Gibco, USA, catalog number: 10270098), and 1% antibiotic/antimycotic (Gibco, USA, catalog number: 15240062) in a humidified incubator set at 37 °C and 5% CO_2_.

### Evaluation of the Effects of Different Condition Media (CM) on T‐Luc EV Production

For CM collection, cells were seeded in a 24‐well plate (Corning, USA) at a density of 50 000 cells per well or 100 000 cells per well after which the growth media was changed to production media 48 or 24 h, respectively. These numbers were chosen so that the cells would be ≈70% confluent at the time of media change from “growth media” to “EV production media.” Unless otherwise mentioned, EV production media were collected for analysis 48 h after being given to the cells.

The different EV production media tested included Opti‐MEM (Gibco, USA, catalog number: 31985070), Opti‐MEM supplemented with 10% FBS, DMEM supplemented with 10% FBS, DMEM supplemented with 10% EV‐depleted FBS (Gibco, USA, catalog number: A2720801), FreeStyle 293 expression medium (Gibco, USA, catalog number: 12338018), virus production serum‐free medium (VP‐SFM) (Gibco, USA, catalog number: 11681020), or MEM media (Gibco, USA, catalog number: 21430079) with and without 10% FBS.

Cell viability and cell count were performed via flow cytometry. After CM was removed, cells were detached with trypsin and washed for 10 min at room temperature (RT) with PBS containing DAPI (4′,6‐diamidino‐2‐phenylindole), and then analyzed with a MACSQuant Analyzer 10 flow cytometer (Miltenyi Biotec, Germany), and FlowJo software (v10, FlowJo LLC) was used to analyze flow cytometric data. Viability is reported as the percentage of DAPI‐negative single cells per total single cell count.

### Evaluation of Chemical Excipients on Level of T‐Luc EV Production in Opti‐MEM CM

For the experiments in which additional reagents were tested in the EV production media, the reagents were introduced systematically after the media‐change step. For the insulin, transferrin, and selenium (ITS) experiments, ITS solution (Gibco, USA, catalog number: 41400045) was added into the production media to the manufacturer's recommended concentration. For the GlutaMAX, fibronectin, globulin, or albumin experiments, GlutaMAX (Gibco, USA, catalog number: 35050061), fibronectin (Sigma‐Aldrich, Germany, catalog number: F1141), globulin (Thermo Scientific, catalog number: 10760815), or albumin (Invitrogen, USA, catalog number: 15561020) were added to Opti‐MEM at the indicated concentrations. In all cases, the CM was collected for analysis 48 h after their addition.

### Challenging the Production T‐Luc EVs in Opti‐MEM CM

The production of the T‐Luc EV was challenged via externally adding known numbers of either HEK wt EVs or MC3‐LNPs. The addition was done to the production media after the culture media‐change step.

HEK wt EVs had been previously collected and stored as described previously.^[^ ^40^ ^]^ Stored HEK wt EVs or LNPs were thawed at RT and numbers were counted using NTA before diluting them in Opti‐MEM to reach desired numbers to be administered to the cells. Opti‐MEM CM was collected for analysis 48 h after their addition.

### CM Collection and Analysis

CM was pre‐cleared first by a low‐speed centrifugation step (500 × *g* for 10 min) to remove dead cells and large cellular debris. CM was then again subjected to centrifugation at 2000 × *g* for 10–20 min to remove other particles and debris. If further processing was conducted, samples were subsequently filtered through a low protein‐binding cellulose acetate membrane filter with a 0.22 µm pore size to remove any remaining larger particles. Collected CMs were then subjected to lysis using Triton X‐100 (Sigma‐Aldrich, Germany), with a final intended concentration of 1% in the CM. For the cells, the lysis was performed using 0.1% Triton X‐100.

After complete lysis, samples (30 µL) were transferred into flat‐bottom white 96‐well microtiter plates (Corning Costar, USA, catalog number: 3917). The lysates were mixed with 25 µL of the luciferase reagent (Promega, Sweden, catalog number: E4550) added by an automated injector. The relative luciferase units (RLU) were determined with a GloMax 96 Microplate Luminometer machine (Promega) with 10 s integration time and 2 s delay between injection. The RLU values from CM were either represented directly or further normalized to either protein content or the RLU values of the cell lysate. Intra‐experimental variability (luciferase substrate age, cell passage, etc.) could contribute to discrepancies in RLUs between experiments.

### Electron Microscopy

Electron microscopy (EM) was performed to visualize HEK293 cell‐derived EVs. EVs were isolated via ultracentrifugation as previously described.^[^ ^52^ ^]^ In brief, CM was collected and centrifuged at 300 x *g* for 5 min and then again at 1200 x *g* for 10 min to remove cell debris, filtered through a 0.22 µm syringe filter, and then ultracentrifuged at 120 000 x *g* for 70 min. The EV pellet was resuspended and were spotted on a glow‐discharged formvar‐carbon type B coated grid (Ted Pella Inc.) and stained with 2% uranyl acetate solution (TAAB Laboratory Equipment, UK, catalog number: U001). The grids were washed with distilled H_2_O and imaged with a FEI Tecnai 10 transmission electron microscope at an accelerating voltage of 100 kV.

### Western Blot

Western blotting (WB) was performed using the iBlot system (Invitrogen, Thermo Fisher Scientific) according to the manufacturer's instructions and as previously described.^[^ ^40^ ^]^ 5 × 10^9^ particles of each sample were mixed with sample buffer (0.5 m ditiothreitol (DTT), 0.4 m sodium carbonate (Na_2_CO_3_), 8% sodium dodecyl sulfate (SDS), and 10% glycerol) and heated at 65 °C for 5 min. The mixture was then loaded onto a NuPAGE Novex 4–12% bis‐tris protein gel (Thermo Fisher Scientific, USA, catalog number: NP0335PK2) and run at 120 V in NuPAGE MOPS SDS running buffer (Thermo Fisher Scientific, catalog number: NP0001) for 2 h. The proteins on the gel were transferred to an iBlot nitrocellulose membrane (Thermo Fisher Scientific) for 7 min using the iBlot system. Membranes were blocked with Odyssey blocking buffer (LI‐COR, USA, catalog number: 927–40100) for 60 min at RT with gentle shaking on a Gel rocker. After blocking, the membrane was incubated 1 h at RT with primary antibody solution (1:1000 dilution for anti‐Tsg101 [Abcam, UK, catalog number: ab30871], anti‐Syntenin [Origene, USA, catalog number: TA504796,]; 1:200 dilution for anti‐CD81 [Santa Cruz Biotechnology, USA, catalog number: sc‐9158]). The membrane was washed with PBS supplemented with 0.1% Tween 20 (PBS‐T) for 5 min, five times and incubated with the corresponding secondary antibody for 1 h at RT (1:15000 anti‐mouse IRDye800CW (LI‐COR, catalog number: 926–32210) to detect Syntenin; 1:15000 dilution anti‐rabbit IRDye800CW (LI‐COR, catalog number: 926–32211) to detect CD81, Tsg101). Membranes were washed with PBS‐T five times within 25 min, one time with PBS and visualized on the Odyssey infrared imaging system (LI‐COR) using Image studio 2.0 (LI‐COR).

### Immunocytofluorescence Staining and Fluorescence Microscopy

HEK293T wt, HEK293T_CD9‐Luc, HEK293T_CD63‐Luc, and HEK293T_CD81‐Luc cells were seeded at 20 000 cells per well in 8‐well glass‐bottom Nunc Lab‐Tek II Chamber Slide (Thermo Scientific, USA). Cells were allowed to adhere for 24 h, washed 1x with PBS, and then fixed with 4% PFA (Fisher Scientific, USA) for 10 min at RT. Both PBS and fixation solutions were warmed up to 37 °C and the pipetting was conducted swiftly to avoid sample drying. Following fixation, cells were permeabilized with 0.05% Triton X‐100 (Sigma‐Aldrich, Germany) in PBS for 5 min at RT. Blocking was performed with 3% bovine serum albumin (Fisher Scientific, USA) in PBS for 2 h at RT. APC‐conjugated primary anti‐CD9 (Miltenyi Biotec, Germany, catalog number: 130‐103‐956), anti‐CD63 (Miltenyi Biotec, catalog number: 130‐100‐182), and anti‐CD81 antibodies (Beckman Coulter, catalog number: A87789) were diluted 1:300 in blocking solution and incubated for 2 h at RT. Stained cells were then washed 3x with PBS. Cells were imaged with an Olympus IX81 fluorescence inverted microscope (Olympus, USA).

### MACSPlex EV Analysis

Bead‐based multiplex EV analysis was performed by flow cytometry using the MACSplex Exosome Kit, human (Miltenyi Biotec) as described previously.^[^ ^47^ ^]^ In brief, a final volume of 60 µL of undiluted CM was loaded onto wells of a pre‐wet and drained MACSPlex 96‐well 0.22 µm filter plate before 10 µL of MACSPlex exosome capture beads were added to each well. Filter plates were incubated overnight at 450 rpm. Beads were washed by adding 200 µL of MACSPlex buffer (MPB) to each well and the filter plate was put on a vacuum manifold with vacuum applied at −100 mBar (Sigma‐Aldrich, Germany) until all wells were drained. For counterstaining of EVs bound by capture beads with detection antibodies, 135 µL of MPB and 5 µL of each APC‐conjugated anti‐CD9, anti‐CD63, and anti‐CD81 detection antibody were added to each well, plates were incubated at 450 rpm for 1 h and then washed by adding 200 µL MPB to each well followed by draining on a vacuum manifold. This was followed by another washing step with 200 µL of MPB, incubation at 450 rpm for 15 min, and draining all wells again on a vacuum manifold. Subsequently, 150 µL of MPB was added to each well, beads were re‐suspended and transferred to V‐bottom 96‐well microtiter plate (Thermo Scientific, USA). Flow cytometric analysis was performed with a MACSQuant Analyzer 10 flow cytometer (Miltenyi Biotec), and FlowJo software (v10, FlowJo LLC) was used to analyze flow cytometric data. Median fluorescence intensities (MFI) for all 39 capture bead subsets were background‐corrected by subtracting respective MFI values from matched non‐EV buffer or media controls that were treated exactly like EV‐containing samples (buffer/medium + capture beads + antibodies).

### Nanoparticle Tracking Analysis

Nanoparticle tracking analysis (NTA) was applied to determine the concentration of all samples. All samples were characterized with a NanoSight NS500 instrument equipped with NTA 2.3 analytical software and a 488 nm laser. At least five 30 s videos were recorded per sample in light scatter mode with a camera level of 11–13. Software settings for analysis were kept constant for all measurements (screen gain 10, detection threshold 7). All samples were diluted in 0.22 µm filtered PBS to an appropriate concentration before analysis.

### RNA Sequencing

Bulk RNA was extracted from cells by resuspending the pellets in 500 µL of TRI Reagent. After 5 min, 100 µL of chloroform was added and the tubes were shaken vigorously for 1 min. After 15 min of incubation, the samples were centrifuged at 12 000 x *g* for 15 min at 4 °C. Then, 300 µL of aqueous phase was mixed thoroughly with 300 µL of isopropanol, 30 µL of 3 m sodium acetate, and 1 µL of pellet paint (Merck, USA) and incubated over night at −20 °C. Subsequently, the samples were centrifuged at 20 000 x *g* for 30 min and the pellets were washed twice with 600 µL of 70% ethanol. After air drying, the pellets were resuspended in 15 µL of elution buffer and RNA concentration was measured using the Qubit RNA high sensitivity assay (Thermo Fischer, USA) according to the manufacturer's instructions.

2 ng of RNA was used as input to the Smart‐seq2 RNA‐sequencing protocol^[^ ^53^ ^]^ and 50 bp single ends were sequenced on an Illumina HiSeq 3000 sequencer. Reads were mapped to the ENSEMBL human transcriptome GRCh37 using Tophat 2.1.1 to generate the read count matrix.

### Bioinformatics Analysis

Hierarchical clustering was performed on sample to sample Pearson correlations based on the most variable genes in the data set expressed above RPKM 4 using the hclust function in R.^[^ ^54^ ^]^ Differential expression analysis utilized the Deseq2 package to compare triplicate samples in R. Up and downregulated genes with an adjusted *p*‐value < 0.001 were then analyzed using Panther Gene Ontology Analysis (panther.org), with a complete set of all genes passing this threshold used as a control group. Gene ontology term fold enrichments for each group were then divided by the enrichment in the control group to arrive at the Term Fold Enrichment displayed.

### Effect of Neutral Sphingomyelinase Inhibitor on TLuc EV Production in Different CM

The ceramide inhibitor GW4869 (Sigma‐Aldrich, Germany) was added to the production media for the final 24 h of cell culture. The working concentration of 7.5 × 10^−6^ m was optimized as the highest working concentration which did not induce cell death (data not shown). CM was collected at the end of the GW4869 treatment period and processed as described above.

### Effect of siRNA Targeting Neutral Sphingomyelinase on TLuc EV Production

siRNA smartpools consisting of four sequences targeting the genes ALIX, SMDP2, and SMPD3 were designed by and ordered from Dharmacon (Horizon Discovery, United Kingdom). Cells were transfected with lipofectamine 2000 at a final concentration of 100 × 10^−9^ m siRNA for the 48 h period before media was changed to production media. siRNA targeting ALIX was used as a control. siRNA‐mediated gene knockdown was confirmed in HEK wt cells with RT‐PCR (Figure S11, Supporting Information). Conditioned media was harvested and subjected to NTA as described above.

### Statistical Analyses

For EV quantitation, results were presented as mean ± SD. For all experiments, the error bars showed the standard deviation of three biological replicate measurements (mean ± SD, *n* = 3 biological replicates). Intra‐experimental variability (e.g., age of reagent or number of freeze–thaw cycles for a given reagent, number of cell passages, pipetting error, etc.) could contribute to discrepancies in RLUs between experiments. Statistical significance (*p < 0.05, **p < 0.01, ***p < 0.001, and ****p < 0.0001) between experimental groups was calculated with two‐way analysis of variance (ANOVA) with no matching and Holm–Sidak correction for multiple comparisons. Significance between differences in EV quantities for a single treatment comparison was calculated with the one‐way ANOVA test with Bonferroni correction for multiple comparisons. Significance tests and data normalization are acknowledged in figure legends.

## Conflict of Interest

The authors declare no conflict of interest.

## Supporting information

Supporting Information

## Data Availability

All sequencing data has been uploaded to the NCBI GEO database under the accession number GSE173474.
