# Supplementary material for: Growth Media Conditions Influence the Secretion Route and Release Levels of Engineered Extracellular Vesicles
Source: Adv Healthc Mater. 2021 Nov 21;11(5):2101658. doi: 10.1002/adhm.202101658 (PMC11469210; doi:10.1002/adhm.202101658)
Supplement: Supplementary file 1 — Supporting Information [file ADHM-11-2101658-s001.pdf]

ADVANCED HEALTHCARE MATERIALS

Supporting Information

for *Adv. Healthcare Mater.*, DOI: 10.1002/adhm.202101658

Growth Media Conditions Influence the Secretion Route and Release Levels of Engineered Extracellular Vesicles

Jeremy P. Bost, Osama Saher*, Daniel Hagey, Doste R. Mamand, Xiuming Liang, Wenyi Zheng, Giulia Corso, Oskar Gustafsson, André Görgens, CI Edvard Smith, Rula Zain, Samir El Andaloussi^, Dhanu Gupta^*

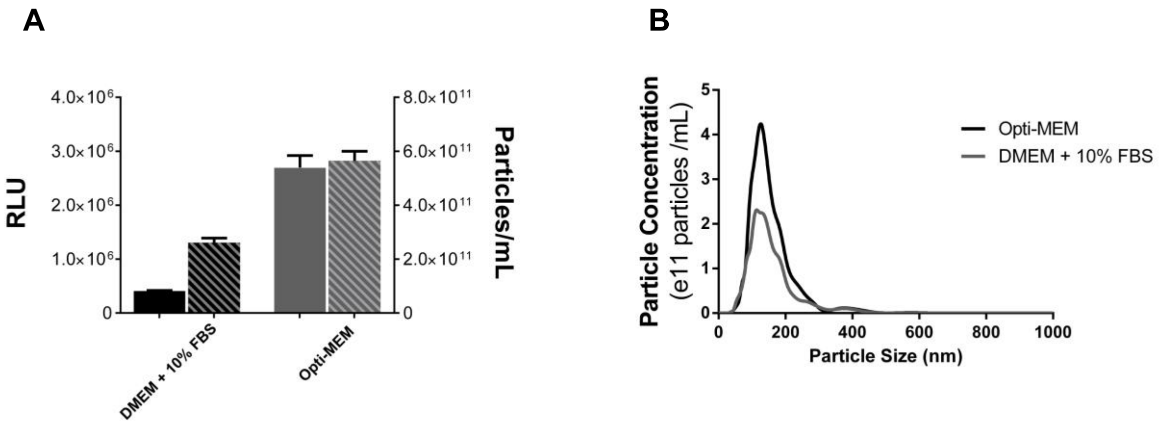

Supplementary Figure 1. NTA characterization of CMs. A) Luciferase activity (left) and nanoparticle concentration (right, striped) of various production media compositions at 48 hr. B) Nanoparticle concentrations and sizes as determined by NTA. All measurements of conditioned media were taken at 48 hr after media change. Luciferase activity is reported as relative luminescence units (RLU). For all experiments, the error bars show the standard deviation of three biological replicate measurements (mean ± SD, *n* = 3). Intra-experimental variability can contribute to discrepancies in RLUs between experiments.

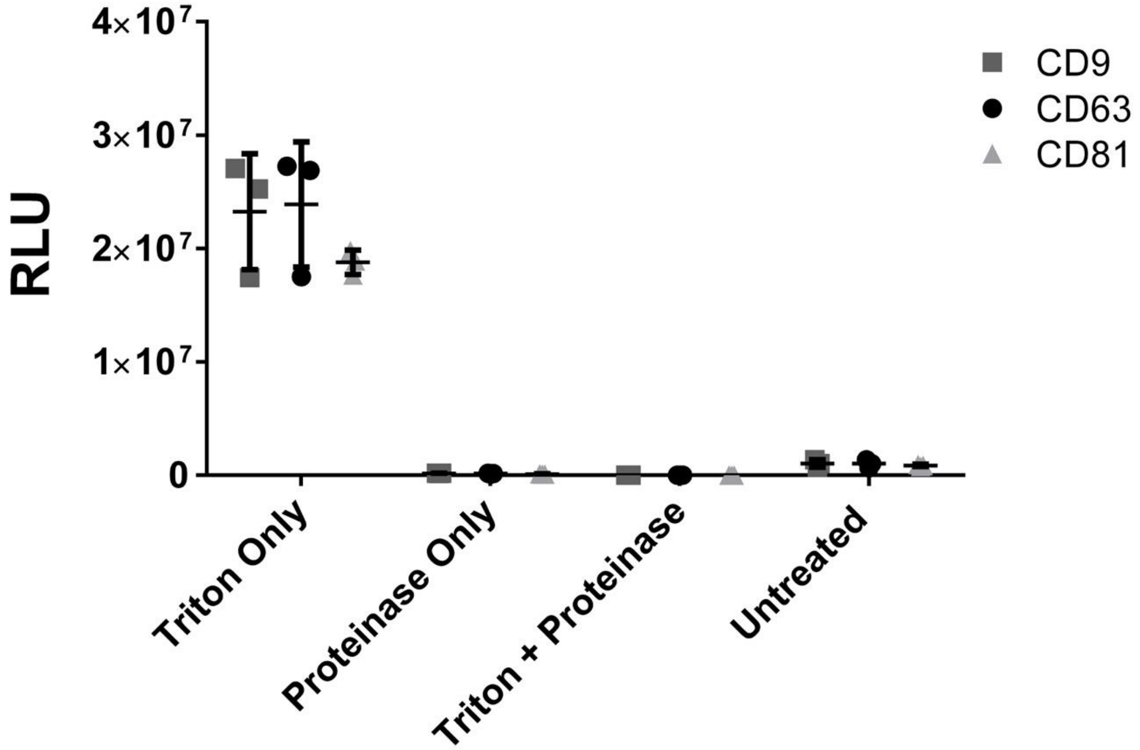

Supplementary Figure 2. CM treatment with Proteinase K. CMs were subjected to treatment either with Triton X-100 (Triton Only), Proteinase K (Proteinase Only), or both (Triton + Proteinase) before luciferase analysis. Luciferase activity is reported as relative luminescence units (RLU). For all experiments, the error bars show the standard deviation of three biological replicate measurements (mean ± SD, n = 3). Intra-experimental variability can contribute to discrepancies in RLUs between experiments.

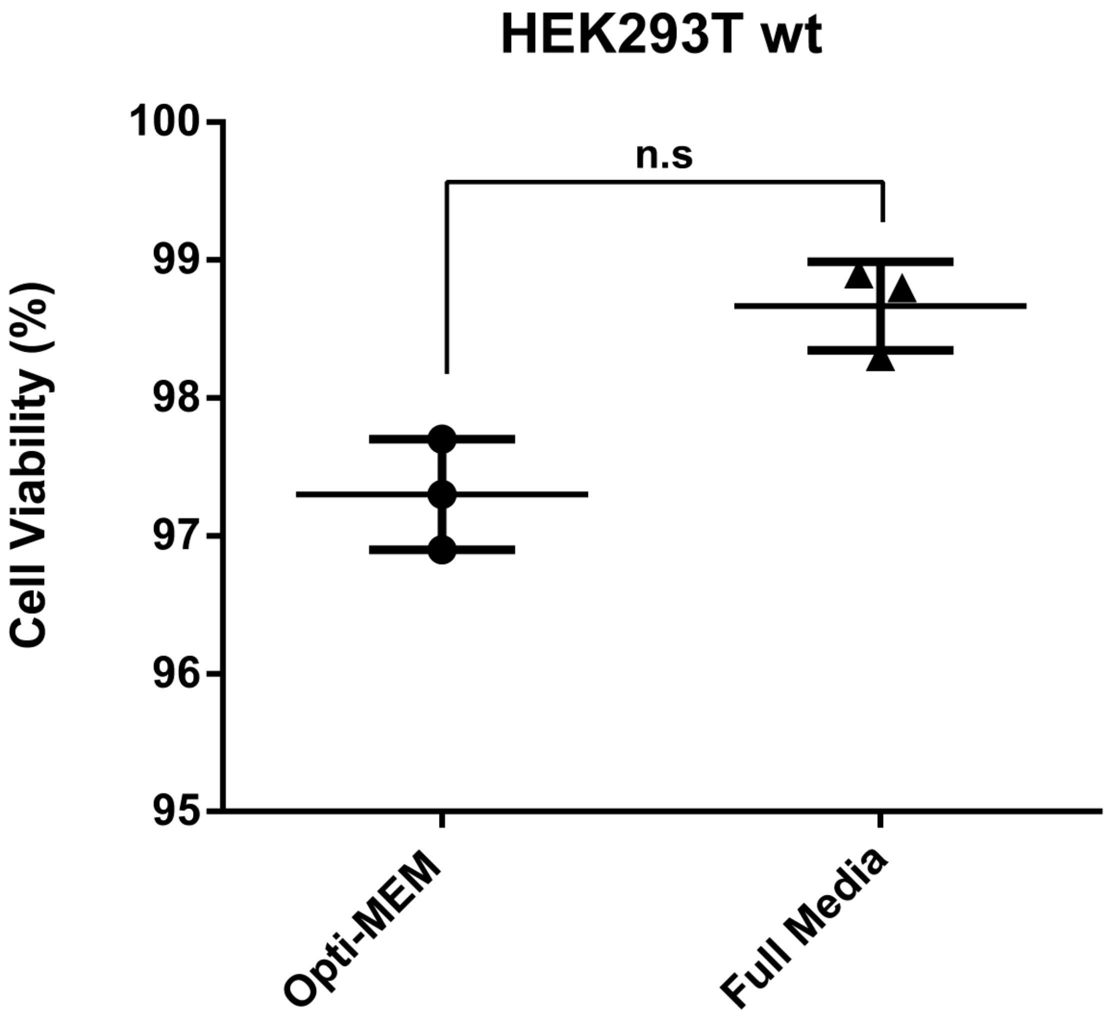

Supplementary Figure 3. HEK wt cell viability after 48 hr in EV-production media. Cell viability measured by DAPI staining and flow cytometry (see Experimental Methods 4.3). Error bars show the standard deviation of three biological replicate measurements (mean ± SD, $n = 3$). Statistical significance (*P < 0.05, **P < 0.01, ***P < 0.001, and ****P < 0.0001) were calculated with Mann Whitney Wilcoxon Test. Intra-experimental variability can contribute to discrepancies in RLUs between experiments.

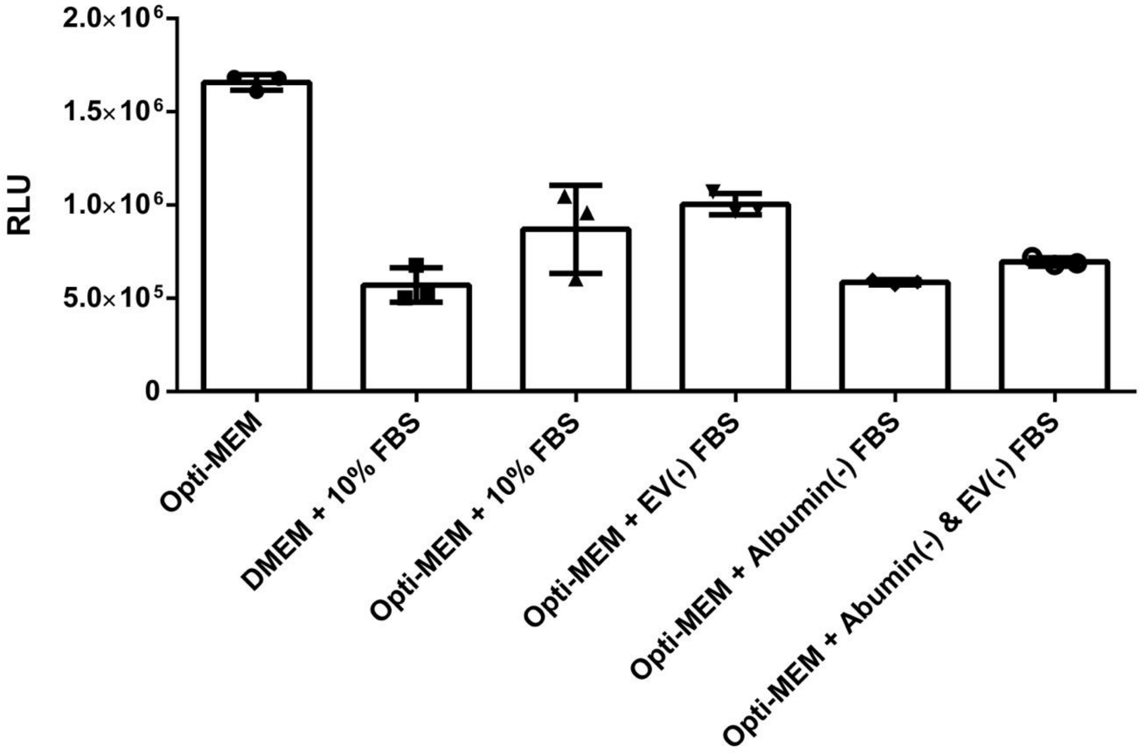

Supplementary Figure 4. Luciferase activity measured from CMs, including Opti-MEM supplemented with 10% EV-depleted FBS (Opti-MEM + EV(-) FBS), Opti-MEM supplemented with 10% albumin-depleted FBS (Opti-MEM + Albumin(-) FBS), and Opti-MEM supplemented with 10% albumin-depleted and EV-depleted FBS (Opti-MEM + Albumin(-) & EV(-) FBS). All measurements of CM were taken at 48 hr after media change. Luciferase values are reported as relative luminescence units (RLU). For all experiments, the error bars show the standard deviation of three biological replicate measurements (mean ± SD, n = 3). Intra-experimental variability can contribute to discrepancies in RLUs between experiments.

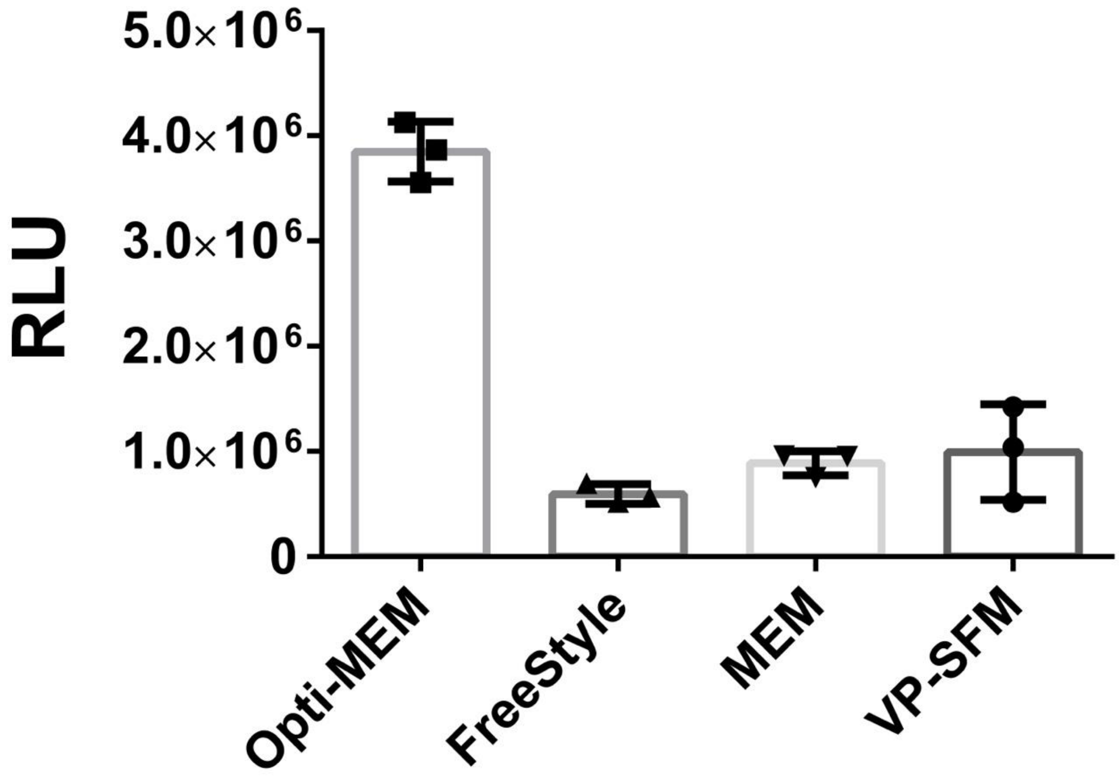

Supplementary Figure 5. Luciferase activity measured from conditioned EV-production media collected from various production media. All measurements of conditioned media were taken at 48 hr after media change. Luciferase values are reported as relative luminescence units (RLU). For all experiments, the error bars show the standard deviation of three biological replicate measurements (mean ± SD, n = 3). Intra-experimental variability can contribute to discrepancies in RLUs between experiments.

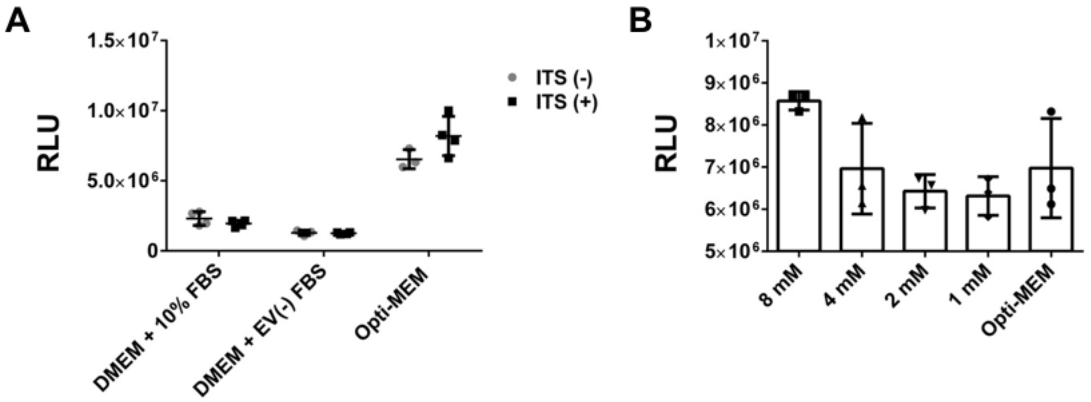

Supplementary Figure 6. Effects of ITS and glutaMAX on EV production. A) Luciferase activity measured from conditioned EV-production media collected from various production media compositions with or without added insulin, transferrin, and selenium (ITS) solution. B) Luciferase activity measured from conditioned EV-production media with added GlutaMAX cell culture reagent. All measurements of conditioned media were taken at 48 hr after media change. Luciferase values are reported as relative luminescence units (RLU). For all experiments, the error bars show the standard deviation of three biological replicate measurements (mean ± SD, *n* = 3). Intra-experimental variability can contribute to discrepancies in RLUs between experiments.

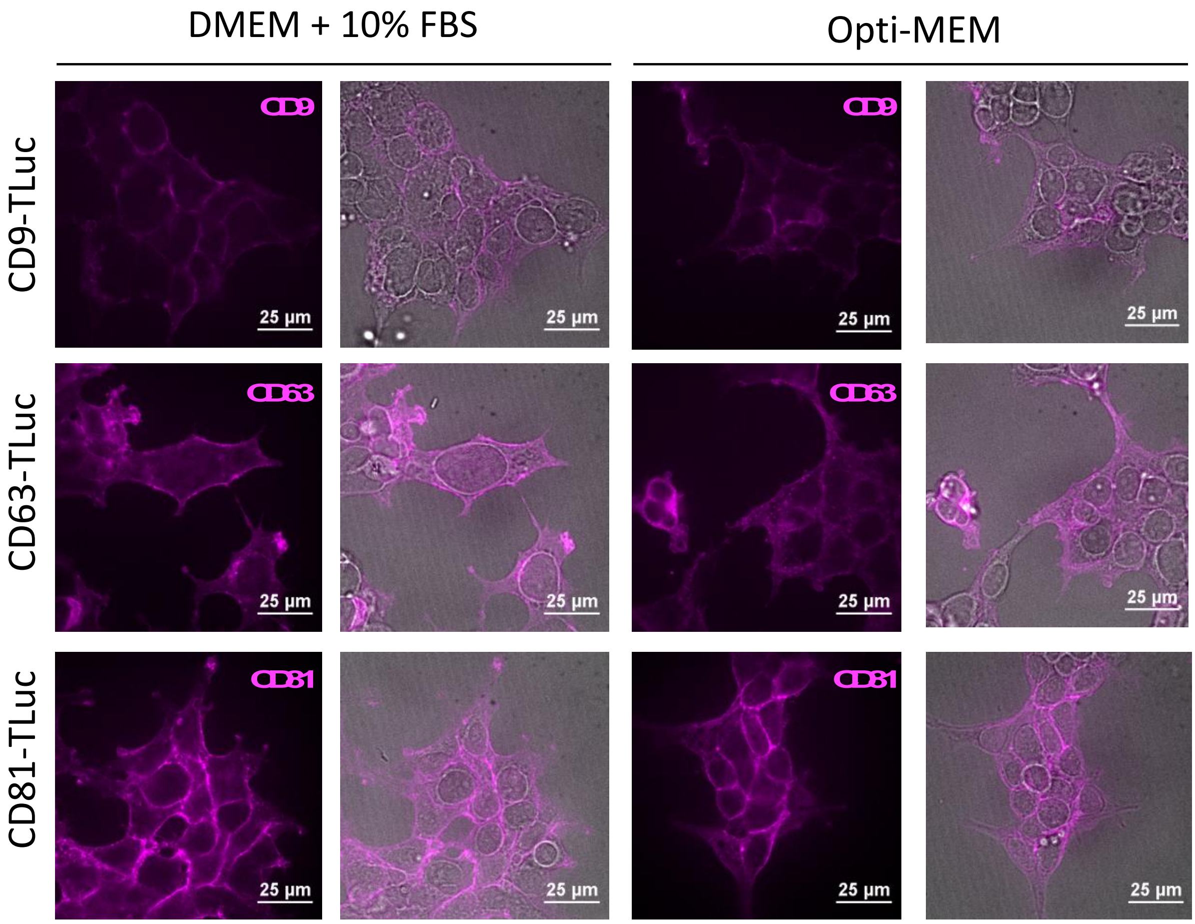

Supplementary Figure 7. Tetraspanin localization in engineered HEK cell lines. A) Fluorescence microscopy visualizing the localization of CD9, CD63, and CD81 their respective engineered EV-producing cells in either DMEM + 10% FBS or Opti-MEM. Brightfield overlay is shown to identify cells. Immunofluorescence staining for CD9, CD63, and CD81 (pink) identifies the localizations of the respective EV-related tetraspanin proteins. Scale bars shown for size.

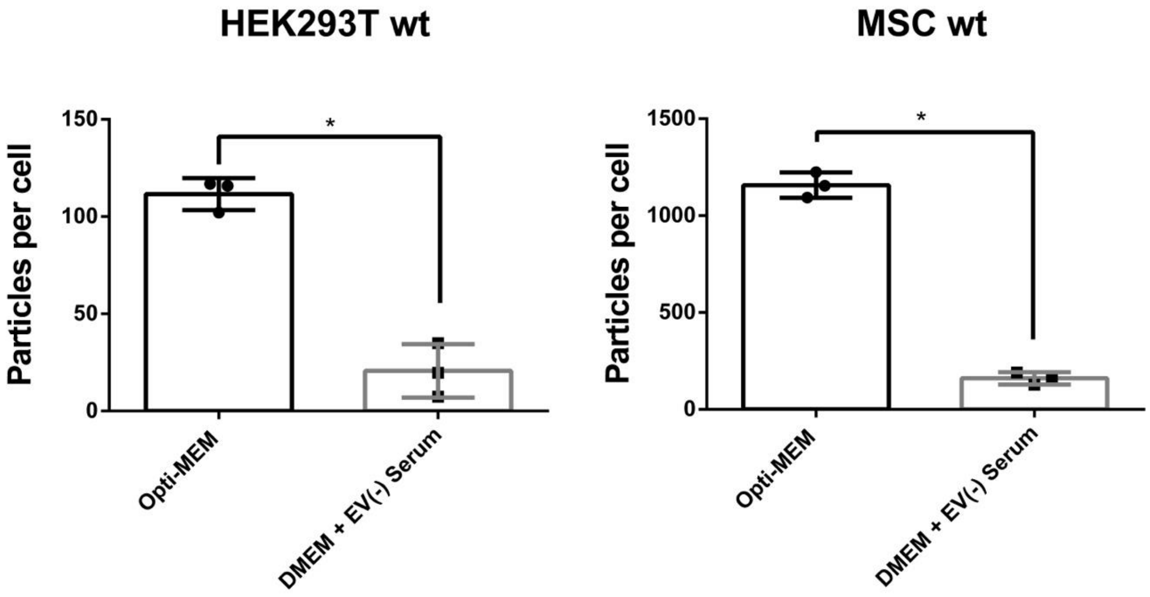

Supplementary Figure 8. Nanoparticle Tracking Analysis of Wild-type CM. Concentration of nanoparticles as reported by NTA in conditioned media from HEK wt or MSC wt cells cultured in Opti-MEM or DMEM supplemented with 10% EV-depleted FBS (DMEM + EV(-) Serum. Particle concentration is normalized to cell count as measured by DAPI staining and flow cytometry. All measurements of conditioned media were taken at 48 hr after media change. NTA particle concentration was normalized to total cell count from each biological replicate. Statistical significance (*P < 0.05, **P < 0.01, ***P < 0.001, and ****P < 0.0001) was calculated with one-way ANOVA test with Bonferroni correction for multiple comparisons. For all experiments, the error bars show the standard deviation of three biological replicate measurements (mean ± SD, *n* = 3).

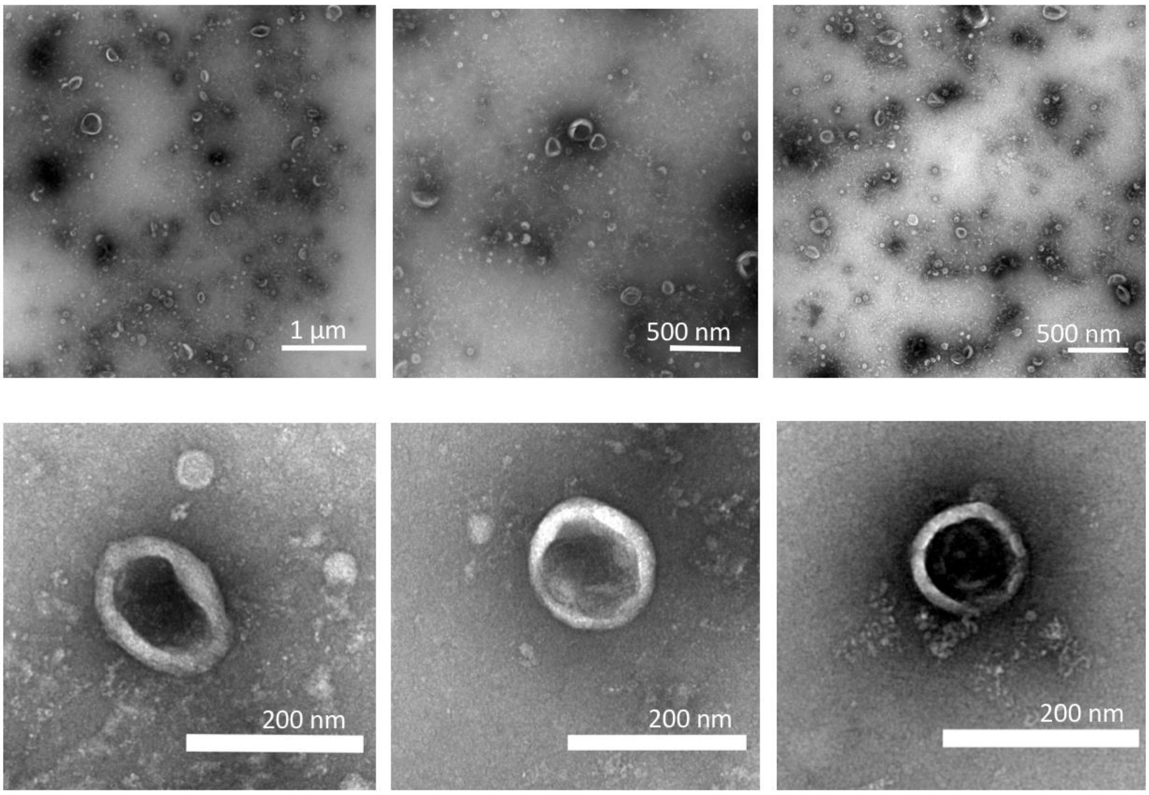

Supplemental Figure 9. Electron microscopy of EVs in Hek wt CM. EM images of UC-isolated EVs showing a wide field (top left, scale bars 1 µm) and enlarged images (top center, top right, scale bars 500 nm; bottom, scale bars 200 nm).

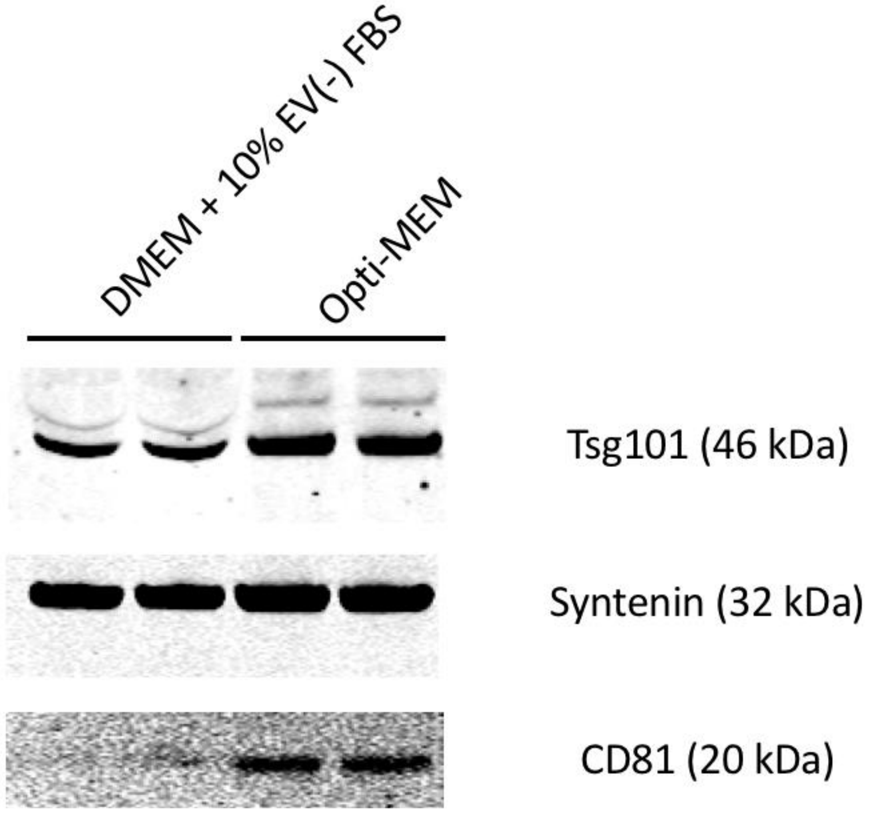

Supplemental Figure 10. Western Blot of EVs markers. WB analysis of HEK wt EVs (5 x 10^9 particles per well) harvested from DMEM + 10% EV(-) FBS EV-production media or Opti-MEM EV-production media. WB was performed in duplicate biological replicates.

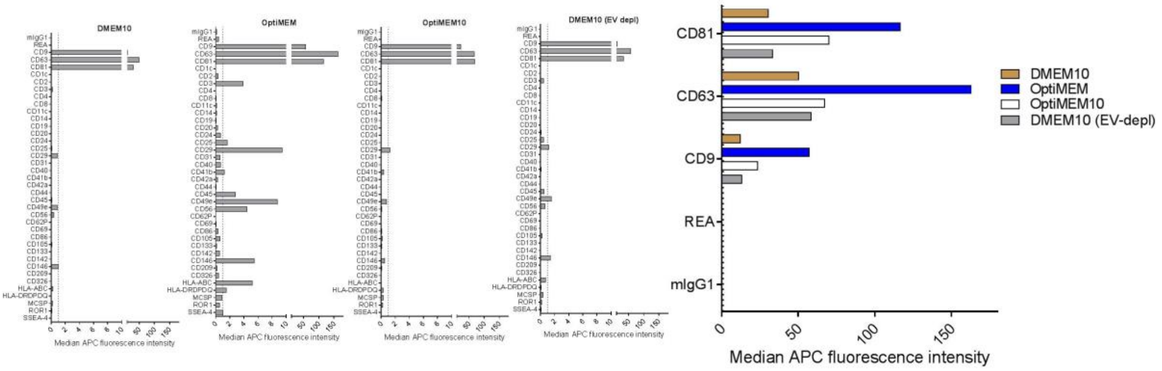

Supplementary Figure 11. MACSPlex EV protein quantitation. Absolute quantitation of 37 EV-related protein levels from conditioned Media containing HEK wt EVs, as reported by MACSPlex. Abbreviations: DMEM10; DMEM containing 10% FBS, OptiMEM; Serum-free Opti-MEM, OptiMEM10; Opti-MEM containing 10% FBS, DMEM10 (EV-dep); DMEM containing 10% EV-depleted FBS.

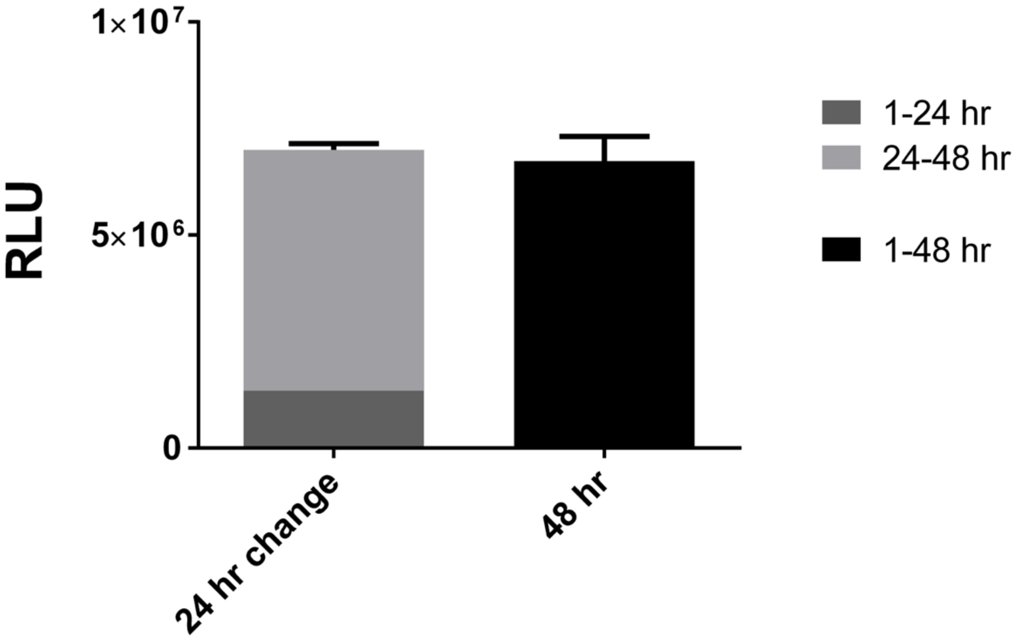

Supplemental Figure 12. EV signal in Opti-MEM CM after 24 hr media change. Cumulative luciferase values from Opti-MEM CM, which had either been changed after 24 hr or acquired after 48 hr. Luciferase values are reported as relative luminescence units (RLU). For all experiments, the error bars show the standard deviation of three biological replicate measurements (mean ± SD, *n* = 3). Intra-experimental variability can contribute to discrepancies in RLUs between experiments.

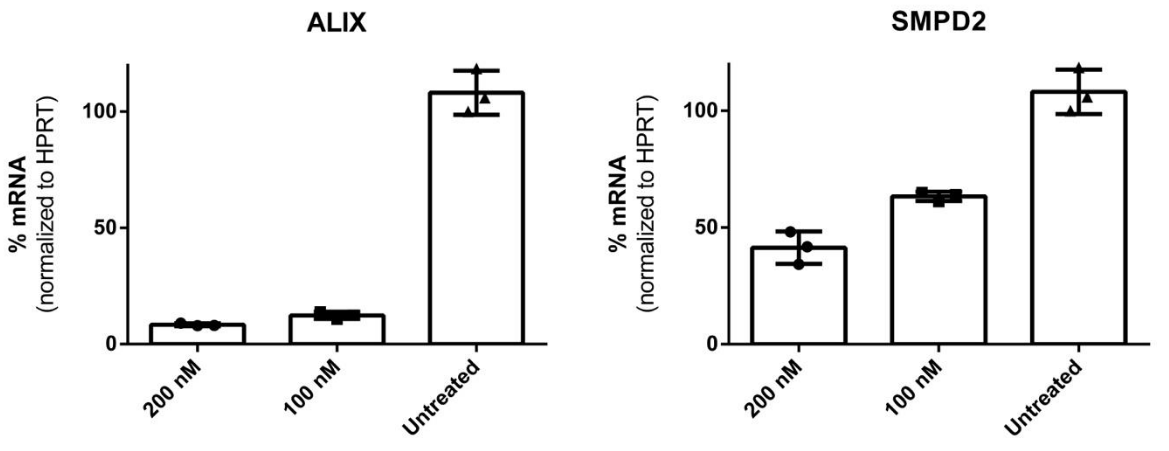

Supplementary Figure 13. siRNA Validation. mRNA levels of siRNA targets ALIX and SMPD2 after treatment with either 100 nM or 200 nM of the respective siRNA. Values are normalized and reported as a percentage relative to transcript copies of HPRT.

Supplementary Table 1. The top 10 upregulated and downregulated GO terms for each pairwise comparison of serum-free Opti-MEM with Opti-MEM + 10% FBS or DMEM + 10% FBS.

Analysis	Gene Ontology Term	P-Value
Opti-MEM upregulated (vs Opti-MEM + 10% FBS)	positive regulation of cellular biosynthetic process (GO:0031328)	3.30E-05
	sphingolipid metabolic process (GO:0006665)	8.25E-05
	sphingolipid biosynthetic process (GO:0030148)	2.02E-04
	amine metabolic process (GO:0009308)	3.71E-04
	membrane lipid metabolic process (GO:0006643)	4.60E-04
	indolalkylamine metabolic process (GO:0006586)	6.19E-04
	positive regulation of nucleic acid-templated transcription (GO:1903508)	6.65E-04
	positive regulation of RNA biosynthetic process (GO:1902680)	6.66E-04
	serotonin biosynthetic process (GO:0042427)	7.07E-04
	positive regulation of RNA metabolic process (GO:0051254)	1.02E-03
Opti-MEM downregulated (vs Opti-MEM + 10% FBS)	cellular metabolic process (GO:0044237)	2.81E-33
	metabolic process (GO:0008152)	1.02E-29
	cellular nitrogen compound metabolic process (GO:0034641)	3.01E-25
	oxidative phosphorylation (GO:0006119)	2.45E-21
	organic substance metabolic process (GO:0071704)	5.80E-21
	nitrogen compound metabolic process (GO:0006807)	2.63E-20
	primary metabolic process (GO:0044238)	3.33E-20
	cellular nitrogen compound biosynthetic process (GO:0044271)	4.56E-20
	protein-containing complex subunit organization (GO:0043933)	1.14E-19
	ATP metabolic process (GO:0046034)	2.54E-19
Opti-MEM upregulated (vs DMEM + 10% FBS)	sphingolipid biosynthetic process (GO:0030148)	2.21E-04

	positive regulation of cellular biosynthetic process (GO:0031328)	5.55E-04
	regulation of axon extension involved in regeneration (GO:0048690)	1.09E-03
	regulation of sprouting of injured axon (GO:0048686)	1.09E-03
	striated muscle cell development (GO:0055002)	1.33E-03
	membrane lipid biosynthetic process (GO:0046467)	1.33E-03
	glycosphingolipid biosynthetic process (GO:0006688)	1.77E-03
	positive regulation of glycolytic process (GO:0045821)	1.77E-03
	cilium-dependent cell motility (GO:0060285)	1.85E-03
	cilium or flagellum-dependent cell motility (GO:0001539)	1.85E-03

Opti-MEM downregulated (vs DMEM + 10% FBS)	cellular metabolic process (GO:0044237)	6.46E-32
	cellular nitrogen compound metabolic process (GO:0034641)	1.72E-29
	metabolic process (GO:0008152)	3.72E-28
	nucleic acid metabolic process (GO:0090304)	3.12E-25
	nucleobase-containing compound metabolic process (GO:0006139)	6.08E-24
	heterocycle metabolic process (GO:0046483)	2.88E-23
	organic substance metabolic process (GO:0071704)	4.00E-23
	primary metabolic process (GO:0044238)	4.00E-23
	cellular aromatic compound metabolic process (GO:0006725)	1.24E-22
	nitrogen compound metabolic process (GO:0006807)	2.21E-22
